# Dynamic regulation of epigenomic landscapes during hematopoiesis

**DOI:** 10.1186/1471-2164-14-193

**Published:** 2013-03-19

**Authors:** Brian J Abraham, Kairong Cui, Qingsong Tang, Keji Zhao

**Affiliations:** 1 Systems Biology Center, NHLBI, NIH, Rockville Pike, Bethesda, MD, USA; 2 Bioinformatics Program, Boston University, Boston, MA, USA

**Keywords:** Epigenetics, Hematopoiesis, Gene expression, Histone modifications

## Abstract

**Background:**

Human blood develops from self-renewing hematopoietic stem cells to terminal lineages and necessitates regulator and effector gene expression changes; each cell type specifically expresses a subset of genes to carry out a specific function. Gene expression changes coincide with histone modification, histone variant deposition, and recruitment of transcription-related enzymes to specific genetic loci. Transcriptional regulation has been mostly studied using *in vitro* systems while epigenetic changes occurring during *in vivo* development remain poorly understood.

**Results:**

By integrating previously published and novel global expression profiles from human CD34+/CD133+ hematopoietic stem and progenitor cells (HSPCs), *in vivo* differentiated human CD4+ T-cells and CD19+ B-cells, and *in vitro* differentiated CD36+ erythrocyte precursors, we identified hundreds of transcripts specifically expressed in each cell type. To relate concurrent epigenomic changes to expression, we examined genome-wide distributions of H3K4me1, H3K4me3, H3K27me1, H3K27me3, histone variant H2A.Z, ATP-dependent chromatin remodeler BRG1, and RNA Polymerase II in these cell types, as well as embryonic stem cells. These datasets revealed that numerous differentiation genes are primed for subsequent downstream expression by BRG1 and PolII binding in HSPCs, as well as the bivalent H3K4me3 and H3K27me3 modifications in the HSPCs prior to their expression in downstream, differentiated cell types; much HSPC bivalency is retained from embryonic stem cells. After differentiation, bivalency resolves to active chromatin configuration in the specific lineage, while it remains in parallel differentiated lineages. PolII and BRG1 are lost in closer lineages; bivalency resolves to silent monovalency in more distant lineages. Correlation of expression with epigenomic changes predicts tens of thousands of potential common and tissue-specific enhancers, which may contribute to expression patterns and differentiation pathways.

**Conclusions:**

Several crucial lineage factors are bivalently prepared for their eventual expression or repression. Bivalency is not only resolved during differentiation but is also established in a step-wise manner in differentiated cell types. We note a progressive, specific silencing of alternate lineage genes in certain cell types coinciding with H3K27me3 enrichment, though expression silencing is maintained in its absence. Globally, the expression of type-specific genes across many cell types correlates strongly with their epigenetic profiles. These epigenomic data appear useful for further understanding mechanisms of differentiation and function of human blood lineages.

## Background

**Figure 1:**
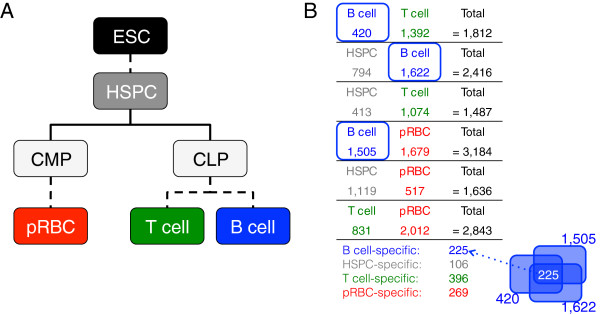
**Many transcripts show cell type-specific expression in hematopoietic subsets.** (**A**) All blood cells derive from hematopoietic stem and progenitor cells (HSPCs) through common progenitors, including the common lymphoid and common myeloid progenitors (CLP, CMP). T and B-cells arise from CLPs, whereas red blood cell precursors (pRBCs) differentiate from CMPs. (**B**) Pairwise comparison of expression profiles from four cell types results in many differentially expressed transcripts but relatively few transcripts with cell type-specific expression. Total: the number of differentially expressed genes between two cell types. The numbers of cell type-specific genes are indicated below the panel.

As every cell type within one organism presumably contains the same genes, differential usage of the genome likely modulates functional differences between cell types. Human blood cells derive from self-renewing hematopoietic stem and progenitor cells (HSPCs) [1]. Differentiating HSPCs undergo progressive loss of differentiation capability in response to environmental conditions, resulting in terminally differentiated blood cells (Figure 1A). Red blood cells derive from HSPCs via a common myeloid progenitor (CMP) and have a nucleated erythrocyte precursor (pRBC) [2-6]. T and B lymphocytes are related immune cell types, both arising through a common lymphoid progenitor (CLP) [7].

Gene expression is dynamically regulated during differentiation, concurrent with epigenetic changes, including both ATP-dependent and post-translational chromatin modification. BRG1-mediated chromatin remodelling contributes to differentiation of both ES cells and HSPCs [8-12]. A variety of histone modifications associates with gene expression. Methylation of H3 lysine 4, catalysed by the MLL family of enzymes [13-15], positively associates with transcription [16-21], whereas trimethylation of H3 lysine 27, mediated by the PRC2 complexes, is associated with silencing [21-25]. Histone variant H2A.Z localizes to active genes and destabilizes nucleosomes [26,27], enhancing access to the DNA and facilitating transcription [28]. Integrating datasets has shown that multiple chromatin states associate with expression status [29,30], and indicated that histone modification patterns may be associated with cell type definition. In particular, coexistence of active and repressive marks at regulatory regions is linked to preparation of differentiation genes [18,31-37]. After differentiation, this bivalency may resolve to allow either activation or repression.

As many previous results were obtained using cell lines cultured *in vitro*, they may not reflect dynamic changes of histone modifications occurring during differentiation *in vivo*. Particularly, although it has been suggested that differentiation genes are bivalently modified in ES cells, it is unclear how bivalency resolves during differentiation *in vivo*. We therefore took advantage of the well-characterized human hematopoietic system and tested the relationship of tissue-specific gene expression to epigenomic changes, and identified common and tissue-specific regulatory elements that may confer tissue-specific transcriptional regulation. Our data indicate that key differentiation genes are primed in progenitor cells by bivalent modification and RNA PolII binding, some even in embryonic stem cells, prior to their expression in the downstream differentiated lineages. Bivalent modification is retained in closely related parallel cell types but is resolved to silent chromatin structure in more remote lineages. Using these epigenomic data, we have identified thousands of common and tissue-specific putative enhancers that might have critical roles in controlling the cell fate decisions during hematopoietic differentiation.

## Results

### Characterization of cell type-specific expression in HSPCs, erythrocyte precursors, B, and T-cells

HSPCs differentiate to generate erythrocytes, B-cells and T-cells. We first sought to characterize cell type-specific (TS) gene expression therein. We calculated gene expression profiles using RNA-Seq [32,38] to identify pair-wise differential expression (Figure 1B). E.g., 794 genes were more highly expressed in HSPCs than in B-cells and 1,622 were more highly expressed in B-cells than in HSPCs. Since B-cells derive from HSPCs, this indicates that 794 and 1,622 genes are repressed and activated, respectively, during differentiation.

To identify TS genes, i.e. those expressed in only one cell type, we intersected those highly expressed in each of the pair-wise comparisons (Figure 1B). Indeed, their overall expression was significantly higher in their specific cell type (Additional file 1: Figure S1A-S1D). These gene lists included the markers used for isolating or defining the populations. E.g., CD133 and CD34 were HSPC-specific; CD4 was T-cell-specific; CD19 was B-cell-specific; and CD36 was pRBC-specific.

In addition to cell surface markers, key transcription regulators were also TS (Additional file 1: Table S1). Among these genes were *PAX5*, the master regulator of B-cell development; *GATA3*, an important regulator of T-cell differentiation; *FOXP3*, a regulator of regulatory T-cells; and *GATA1*, a master regulator of RBC development. That these genes of known type-specific importance were called type-specific shows that our tests extract high-confidence type-specific transcripts.

KEGG pathway analyses of TS genes showed associations with expected functions. The B-cell receptor signaling and IgA production pathways were enriched in B-cell-specific genes (Additional file 1: Figure S2C); T-cell-specific genes were similarly overrepresented in the T-cell receptor pathway, as well as in immunodeficiency and cell adhesion molecules (Additional file 1: Figure S2B). Although erythrocyte transcripts, including many versions of hemoglobin, were specifically expressed in pRBCs, these genes were enriched in KEGG pathways of more general biochemical function (Additional file 1: Figure S2D).

### Chromatin environment at transcription start sites

**Figure 2:**
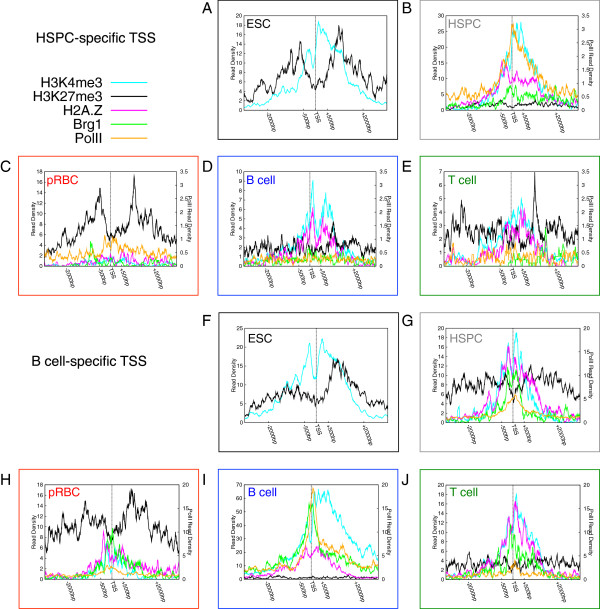
**Chromatin profiles around TSSs of type-specific genes show extensive priming in progenitor cells during differentiation.** (**A**) – (**E**) Histone modification, BRG1 binding and RNA Polymerase II binding profiles around TSSs of HSPC-specific genes are displayed for (**A**) ESC, (**B**) HSPC, (**C**) pRBC, (**D**) B-cell, and (**E**) T-cell. (**F**) – (**J**) Histone modification, BRG1 binding and RNA Polymerase II binding profiles around TSSs of B-cell-specific genes are displayed for (**F**) ESC, (**G**) HSPC, (**H**) pRBC, (**I**) B-cell, and (**J**) T-cell.

To investigate relationships between gene expression and chromatin environment in progenitor and differentiated cells, we profiled genome-wide distributions of several histone modifications (H3K4me1, H3K4me3, H3K27me1, and H3K27me3), histone variant H2A.Z, chromatin remodeler BRG1, and RNA PolII using ChIP-Seq (see methods). We then plotted tag densities surrounding transcription start sites (TSSs) of HSPC-specific genes (Figure 2A - 2E). As expected for expressed genes, PolII was highly enriched in these promoters in HSPCs (Figure 2B) while no appreciable binding of PolII to these promoters occurred in pRBCs (Figure 2C), B-cells (Figure 2D) and T-cells (Figure 2E). Similarly, BRG1 was enriched in these promoters only in HSPCs. H3K4me3 and H2A.Z were enriched in promoters of HSPC-specific genes not only in HSPCs but also in T- and B-cells, but and less so in pRBCs. While nominal promoter H3K27me3 signals were detected in HSPCs, they were highly enriched in the other three cell types, confirming that gaining H3K27me3 at these HSPC-specific genes during differentiation is associated with silencing (compare Figure 2B to 2C, D and E). To investigate priming of HSPC-specific genes, we investigated their histone modification status in embryonic stem cells (ESCs) (Figure 2A) using published datasets [39]. In ESCs, these promoters were enriched in H3K4me3, indicating preparation for their expression in HSPCs. However, the promoters were also marked with H3K27me3, which was lost at the HSPC stage, indicating further that their expression is associated with loss of H3K27me3.

To expand these observations to further differentiated cells, we profiled chromatin proteins at B-cell-specific genes (Figure 2F-J). We note that, although these genes were not yet expressed in HSPCs, they had been associated with high levels of H3K4me3, H2A.Z and BRG1 (Figure 2G), and with high levels of H3K4me3 in ESCs (Figure 2F), suggesting that priming of these genes occurred before their expression in the differentiated cells. Binding of PolII was also detected on 17% of these genes in HSPCs, suggesting some were poised for expression. T-cells and B-cells descend from similar progenitors. B-cell-specific genes retained substantial signals of H3K4me3, H2A.Z and BRG1 in T-cells (Figure 2J), suggesting that these genes may have not been fully silenced and may retain expression potential.

**Figure 3:**
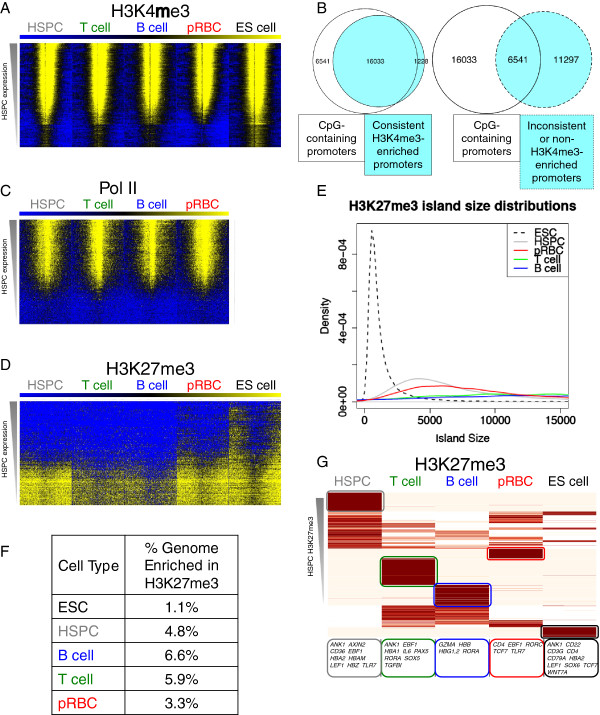
**H3K4me3 profiles are consistent but H3K27me3-enriched regions change drastically during differentiation.** (**A**) Heatmaps of H3K4me3 around TSSs sorted into 200 groups by HSPC expression show stable marking of the most highly expressed genes across all cell types and a depletion directly at the TSS. (**B**) Promoters consistently marked by H3K4me3 in the four hematopoietic cell types contain a CpG island more often than those that are inconsistently or not marked by H3K4me3. (**C**) RNA PolII heatmaps around TSSs sorted into 200 groups by HSPC expression show PolII binding at the most highly expressed genes directly at the TSS. (**D**) H3K27me3 heatmaps around TSSs sorted into 200 groups by HSPC expression show enrichment in the lowest expressed genes. (**E**) The distributions of the sizes of H3K27me3-enriched regions in ESC (black dotted), HSPC (grey), pRBC (red), T-cell (green), and B-cell (blue) show that H3K27me3-enriched regions grow in size in differentiated cell types in comparison with ESCs and HSPCs. (**F**) Percentages of the genome falling in SICER islands calculated for H3K27me3. (**G**) Most of the H3K27me3-enriched regions are cell type-specific. Regions of H3K27me3 enrichment were unified across all five cell types, broken evenly into ≤ 2kbp fragments, clustered by their H3K27me3 read counts, and displayed as a heatmap.

The above analyses of cell type-specific genes indicate that, while H3K4me3 signals are generally correlated with gene expression though are sometimes present even in non-expressing cells, changes in H3K27me3 are more closely related to inactive expression states in different cell types. To extend this observation, we sorted all genes into 200 groups according to their RPKM in HSPCs and averaged ChIP-Seq read densities of these two modifications across a 6kb region surrounding TSSs (Figure 3A and 3D). Indeed, average profiles of H3K4me3 at genes highly expressed in HPSCs were consistent across all cell types, which may be related to the H3K4me3 signals at house-keeping genes, and levels of H3K4me3 positively associated with transcription (Figure 3A). DNA sequence analysis indicated that promoters associated with constitutive H3K4me3 were enriched in CpG islands (Figure 3B**)**. Of 17,261 promoters consistently enriched in H3K4me3 in the four cell types, 93% also contained a UCSC-defined CpG island [40]; merely 37% of promoters with no or inconsistent H3K4me3 enrichment contained a CpG island. The correlation between H3K4me3 and CpG islands was observed previously [31], however the additional correlation with H3K4me3 consistency was not. We also noted that all cell types showed enrichment of H3K4me3 upstream and downstream of a depletion at the TSS, and that H3K4me3 extended further downstream of the TSS than upstream. This pattern was similar to PolII at the same TSS groups, sorted and calculated in the same manner (Figure 3C). Despite fewer PolII-bound than H3K4me3-associated promoters, the majority of TSS groups showed some presence of PolII positively correlating with expression. H3K27me3 signals were mainly enriched in gene groups with low expression and occupied much of the interrogated region, contrasting with H3K4me3 and PolII (Figure 3D).

The widespread signals of H3K27me3 suggest that it occupies broad domains, consistent with previous reports [32,41]. To examine the size of H3K27me3 domains during differentiation, we compared the sizes of islands across the four cell types. The distribution of H3K27me3 island sizes peaked at around 1.5kb in ESCs, 4.2kb in HSPCs, and increased substantially in pRBC, T and B-cells (Figure 3E), demonstrating that differentiation results in larger regions of H3K27me3 enrichment. We also found that H3K27me3 marks larger genomic regions in downstream cell types (Figure 3F).

To further investigate changes and growth of H3K27me3-modified regions during differentiation, we performed a clustering analysis of regions enriched in H3K27me3 in these cell types (Figure 3G). We evenly split each united region into ≤ 2kbp sections, creating 222,914 regions enriched in H3K27me3 in at least one cell type. We found that most of the H3K27me3-enriched regions changed during differentiation, underscoring the variability of H3K27me3 targeting. In fact, most regions enriched in H3K27me3 in downstream cell types were not enriched in HSPCs, and there was no cluster enriched in H3K27me3 in all five cell types. There were many regions that specifically gained H3K27me3 in either the T, B, or pRBC stages, but relatively few that became enriched in all three. After assigning regions to their nearest gene within 20kb, we noted that regions enriched in H3K27me3 in a single cell type coincided with genes with non-hematopoietic function (DATA NOT SHOWN). Genes nearest to H3K27me3-enriched regions in pRBCs alone were enriched in cytokine-cytokine receptor interaction genes (DATA NOT SHOWN), indicating repression of immunological pathways in this myeloid lineage. Several type-specific genes coincided with regions enriched in H3K27me3 in one cell type, as highlighted in Figure 3G, indicating silencing of signature gene expression. Curiously, 80% of genes associated with the cluster of regions enriched solely in HSPCs (upper leftmost cluster) remained silent in downstream cell types, even in the absence of H3K27me3. This indicates that, while H3K27me3 enrichment is indicative of repression, its absence is not necessarily indicative of activation as subsequent factors may maintain silencing and/or activation factors may not be present.

The dynamic regulation of H3K27me3 during differentiation is exemplified at the *HOXA* and *HOXB* developmental gene loci, which show initial enrichment followed by substantial loss then reconstitution at select genes, corresponding with their expression [31,32] (Additional file 1: Figure S3). This supports widespread H3K27me3 signals together with loss of active histone modifications stabilizing a silent chromatin conformation after differentiation.

### Bivalent marking of promoters in HSPC and resolutions upon differentiation

**Figure 4:**
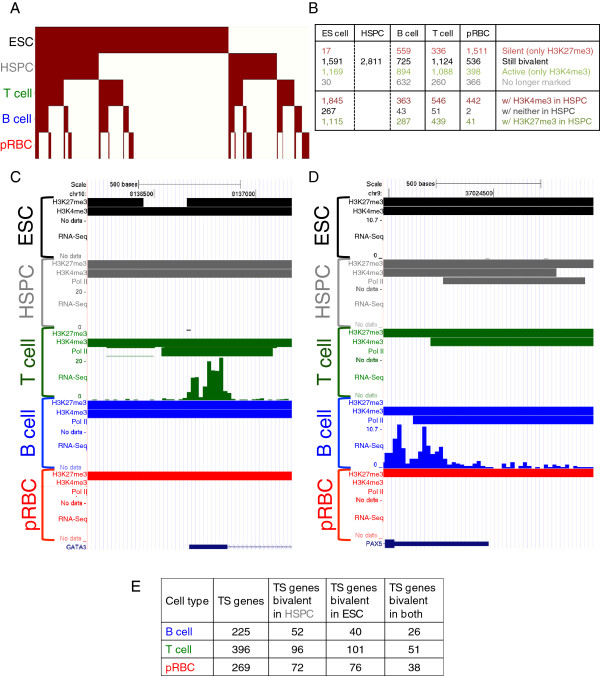
**Bivalent priming of TSSs is prevalent and its resolution varies during differentiation.** (**A**) Resolution and formation of bivalency during differentiation. Each column represents a gene bivalent in any of our cell types and is colored in the cell types in which it is bivalent. Columns/genes were grouped by their bivalency across cell types. (**B**) Bottom panels represent genes bivalently marked outside the HSPC stage. The number of genes possessing H3K4me3 but lacking H3K27me3 in HSPCs (red), possessing H3K27me3 but lacking H3K4me3 in HSPCs (green), and possessing neither in HSPCs (black) are shown. (**C**) The T-cell regulator *GATA3* shows bivalent priming and resolution. In ESCs (black), HSPCs (grey) and B-cells (blue) the *GATA3* promoter (TSS +/− 0.5kbp) is enriched with H3K4me3 and H3K27me3 and is not transcribed. In pRBCs (red), only H3K27me3 is found. In T-cells (green), *GATA3* is bound by PolII and is transcribed. (**D**) The B-cell master regulator *PAX5* is bivalently marked in ESCs (black), HSPCs (grey) and T-cells (green). It is bound by PolII in HSPCs as well. In pRBCs (red), H3K4me3 is lost, leaving only H3K27me3. In B-cells (blue), *PAX5* is enriched in H3K4me3, bound by PolII, and uniquely expressed. (**E**) Genes specifically expressed in downstream lineages are bivalently prepared in HSPC and ESC.

The above indicates that genes specifically expressed in downstream cell types are associated with active chromatin marks, e.g. H3K4me3, in upstream cell types, although they are not expressed and may be previously marked with silencing H3K27me3. The coexistence of H3K4me3 and H3K27me3, termed bivalent modification, was discovered in ES and T-cells and proposed as a preparation for genes to be expressed in response to environmental cues [18,31-34]. We sought to understand differentiation-coupled bivalency resolution by constructing a heatmap of promoter bivalency status (Figure 4A). Most of the 5,345 promoters showing bivalency in any of our five cell types were bivalent in ESCs and most of these lost bivalency in downstream cell types. Stem cells had more bivalent genes than the more committed cell types, but several genes developed bivalency in downstream cell types.

To find examples of genes showing bivalency and resolution, we investigated the bivalent promoters in our cell types (Additional file 1: Figure S4). A minor fraction of these was bound by PolII. The Venn diagram shows different partitioning of bivalent promoters in various cell types (Additional file 1: Figure S4). Several important genes with TS function were bivalently marked in progenitor or parallel non-expressing cells. The zinc finger transcription factor *GATA3* is essential for development of T-cells [42]. Our data indicate that, although the *Gata3* promoter was associated with H3K27me3 in HSPCs and ESCs, consistent with its silent state, it also was marked by H3K4me3, suggesting that the gene is primed for expression in progenitor cells (Figure 4C, Additional file 1: Figure S5). When expressed in T-cells, H3K27me3 disappeared from the *GATA3* gene, accompanied by appearance of PolII. We note that both the H3K4me3 and H3K27me3 signals remained in B-cells (Figure 4C in blue), while the H3K4me3 signal largely disappeared in pRBCs (Figure 4C in red).

Similarly, the gene for paired box transcription factor *PAX5*, the master regulator of B-cells, was associated with H3K4me3 and PolII, but not H3K27me3 in B-cells (Figure 4D**,** Additional file 1: Figure S5). Our data indicate that the *PAX5* promoter is bivalent and bound by PolII in HSPCs, and bivalent in ESCs, suggesting that the gene has been prepared for expression (Figure 4D in grey). Interestingly, *PAX5* was still bivalent in T-cells, although the PolII signal disappeared (Figure 4D in green). In contrast, *PAX5* contained only H3K27me3 in pRBCs, indicating that the gene is fully silenced therein (Figure 4D in red).

In addition to the regulators discussed above, several other factors were bivalent in different combinations of the cell types (Additional file 1: Figure S4). *CD8A*, a defining marker of CD8+ T-cells, was bivalent in all except the CD4+ T-cells, where it was monovalently active, indicating a potential for plasticity among T-cell subsets. Another factor, *TCF7*, which is essential for T-cell development as well as regulation of the self-renewal/differentiation switch [43], was bivalent in HSPCs and ESCs, as was cardiac development regulator *GATA5*. In addition to cell type-related genes, several chromatin genes were bivalent. Notably, all genes highlighted in Additional file 1: Figure S4, except the histone genes and *HDAC8*, were also bivalent in ESCs.

Globally, we found 2,811 promoters bivalent in HSPCs and calculated their bivalency status in each differentiated cell type (Figure 4B). Curiously, 20-40% of these promoters remained bivalent in differentiated cells, some of which may be important for further differentiation as previously seen for T helper cells [44]. Generally, more HSPC bivalent promoters lost H3K27me3 than lost H3K4me3, except in pRBCs. We also investigated promoters that became bivalent in downstream cell types and analyzed their status in HSPCs. Strikingly few promoters had had neither mark in HSPCs. We noted that pRBCs, which differ from the other cell types as they were differentiated *ex vivo* from HSPCs [32], showed slight differences in ratios of bivalent HSPC promoter resolutions—many more lost H3K4me3 than remained bivalent, and fewer newly bivalent promoters had had H3K27me3 enrichment in HSPCs. This may reflect the degree of commitment of the cell type, as B and T-cells differentiate further. Although many bivalent promoters lost H3K27me3 enrichment in downstream cell types, most did not gain appreciable expression as a result (DATA NOT SHOWN). This further indicates a role for other factors maintaining silencing in the absence of H3K27me3. Many more genes were bivalently marked in ESCs than HSPCs. Nearly 40% of promoters bivalent in HSPCs were also bivalent in ESCs. Surprisingly few promoters bivalent in HSPCs were marked exclusively by H3K27me3 in ESCs, further indicating that genes are primed for expression notably early in development.

We also examined bivalent priming of TS genes (Figure 4E). These results indicate that many genes expressed in downstream cell types are primed for expression in progenitor cells. Several TS genes with transcription factor activity were bivalent in HSPCs (Additional file 1: Figure S6), underscoring the importance of bivalency in controlling expression of lineage regulators. However, priming of these genes with PolII was not extensive in HSPCs. Several of these genes had multiple isoforms, which showed differences in bivalency at their discrete promoters, while all isoforms were type-specific by their expression. This suggests a role for chromatin in mediating isoform-specific expression.

### Prediction and analysis of distal regulatory elements from chromatin profiles

**Figure 5:**
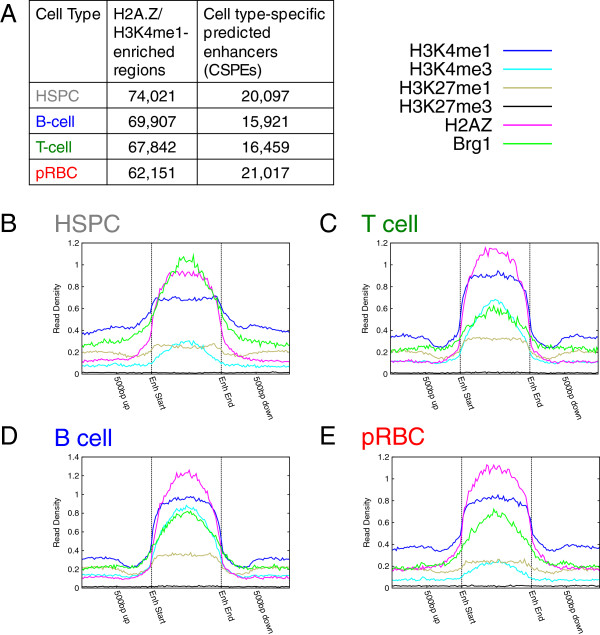
**The chromatin environment at core potential enhancers (CPEs) remains stable across cell types.** (**A**) Counts of H2A.Z/H3K4me1-enriched regions and CSPEs in each cell type. (**B**) – (**E**) Chromatin profiles of size-normalized CPEs in (**B**) HSPCs, (**C**) T-cells, (**D**) B-cells, and (**E**) pRBCs all show enrichment of H3K4me1 and H2A.Z by definition. They are also all enriched in Brg1, H3K4me3, H3K27me1, and PolII but lack H3K27me3 enrichment.

Distal regulatory elements play critical roles in lineage fate decisions and may contain cell type-specific chromatin modification patterns [29,45,46]. Chromatin modifications and binding of chromatin-modifying enzymes indicate the presence of potential regulatory elements [47,48]. Enrichment of H3K27ac and binding of p300 are detected at active enhancers [45,46,49]. In order to completely survey both active and poised enhancers, we used enrichment of H2A.Z and/or H3K4me1 to predict genomic regions outside of a promoter (see methods) as potential enhancers, since previous studies reported that enhancers are associated with H3K4me1 [50] and the histone variant H2A.Z [27,51]. Among enriched sites in each cell type **(**Figure 5A second column), we identified 11,283 regions enriched in these marks across all four cell types (core potential enhancers or CPEs). We also found regions enriched in these marks in only one cell type **(**cell-specific potential enhancers or CSPEs) **(**Figure 5A third column). Among the regions enriched in the marks were several from the hemoglobin beta chain gene locus, which have been reported as functional enhancers (Additional file 1: Table 2). Other known enhancers predicted by our method included the first intron of the *RUNX1* gene, which contains a functional enhancer, a *PAX5* enhancer, and the *PLAT* enhancer. These results indicate that our method is successful in finding regions with enhancer activity.

**Figure 6:**
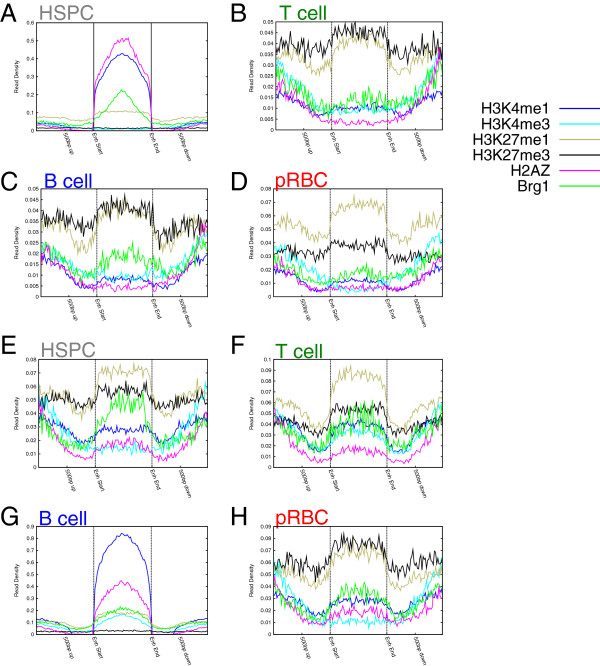
**The chromatin environment of cell-specific potential enhancers (CSPEs) varies greatly during differentiation.** (**A**) – (**D**) Chromatin profiles around size-normalized HSPC-specific CSPEs are displayed for (**A**) HSPCs, (**B**) T-cells, (**C**) B-cells, and (**D**) pRBCs. (**E**) – (**H**) Chromatin profiles around size-normalized B-cell-specific CSPEs are displayed for (**E**) HSPCs, (**F**) T-cells, (**G**) B-cells, and (**H**) pRBCs.

To examine chromatin modifications at these predicted enhancers, we plotted ChIP-Seq reads at the 11,283 CPEs (Figure 5B-E). CPEs were consistently enriched in our defining marks (H2A.Z and H3K4me1) and active marks (H3K4me3, PolII, BRG1), but were depleted of H3K27me3. In contrast to CPEs, HSPC CSPEs exhibited enhancer-like chromatin only in HSPCs (Figure 6A). In other cell types, HSPC CSPEs lost active H2A.Z and H3K4me1 marks, and gained H3K27me3. Curiously, H3K27me1, whose function is poorly understood, was present at HSPC CSPEs in all cell types (Figure 6B, C, D). Similarly, B-cell CSPEs showed elevated active marks in B-cells (Figure 6G), and lost active marks but gained the repressive H3K27me3 mark in other cell types (Figure 6E, F, H). We note that B-cell CSPEs were already associated with elevated levels of BRG1 binding in HSPCs (Figure 6E), suggesting that chromatin remodeling by BRG1 may be required for subsequent establishment of B-cell-specific enhancers, consistent with previous observations in erythrocyte differentiation [8]. The presence of poorly understood H3K27me1 is puzzling, as previous analyses have shown that H3K27me1 in gene bodies positively correlates with expression [16], and that it may show some enrichment in enhancers [52]. That these TS elements contain repressive marks in the other cell types is logical, since, combined with the lack of active marks, this could result in silencing of target genes.

**Figure 7:**
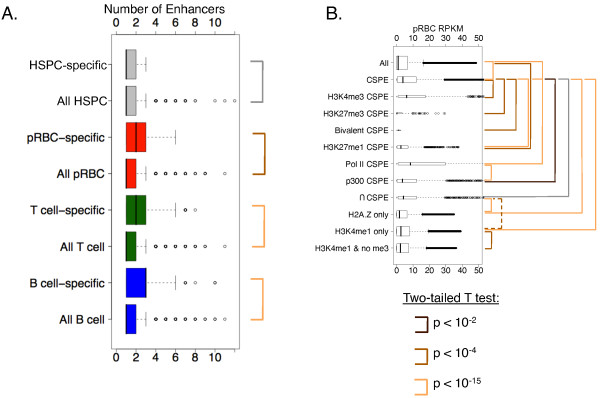
**The presence of predicted enhancers and their chromatin environment both affect target gene expression.** (**A**) Cell type-specific genes have significantly more CSPEs in the corresponding cell type. A CSPE is assigned to its nearest gene within +20kb or –20kb outside of the gene region and 500bp outside a promoter. The number of CSPEs from the corresponding cell type associated with cell type-specific genes (top) is significantly higher than the number of CSPEs associated with all genes with RPKM > 0 (bottom) in pRBCs (red), T-cells (green), and B-cells (blue). (**B**) Genes associated with CSPEs are significantly more highly expressed than predicted (All vs. CSPE p < 2.2 × 10^-16^). Potential target genes of CSPEs are sorted based on the chromatin environment at CSPEs or association with Brg1, PolII and p300 and their expression levels are compared to all genes. Significant differences are indicated by colored lines. See Additional file 1 for p-values and discussion.

To investigate how predicted enhancers correlate with the expression of nearby genes, we compared the counts of CSPEs associated with TS genes and versus CSPEs associated with all genes with RPKM greater than 0 (Figure 7A). We found that type-specific expression tended to associate with more CSPEs, indicating that these CSPEs tend to be expression activators.

To associate histone marks at CSPEs with expression, we compared the distributions of pRBC expression of genes associated with pRBC-specific CSPEs enriched in different marks (Figure 7B). Genes associated with a CSPE were more highly expressed than all genes (p < 2.2 × 10^-16^), further indicating that these elements positively correlate with expression. Genes associated with a PolII-bound CSPE tended to show higher expression (vs. all genes p < 2.2 × 10^-16^, vs. CSPE-containing genes p < 2.2 × 10^-16^) (Figure 7B). PolII at these potential enhancers may result from direct binding of PolII to enhancers or indirectly from enhancer-promoter interactions. Several histone modifications at CSPEs were tested for their impact on expression of their nearest genes (for full discussion, see Additional file 1**)**, showing strong evidence that chromatin environments of these elements correlates strongly with expression.

## Discussion

Epigenomic changes during development are crucial factors that explain type-specific function [16,18,31,32,42,45,46,50], [53]. Here we compared expression and epigenomic differences among human hematopoietic progenitors and downstream lineages. Our results indicate that genes activated in downstream lineages are marked by bivalent histone modifications and some are also bound by RNA PolII in progenitor cells. Bivalent modifications resolve in specific lineages but may remain in parallel differentiated lineages, depending on the relative distance of the cell types. We also predict enhancer-like elements that may contribute to the observed tissue-specific expression.

Co-existence of active and inactive chromatin modifications at promoters has been detected in various cell types [18,31-33,54]. Genes with such bivalency preferentially show tissue-specific expression in downstream cell types [55]. These promoters generally resolve to monovalency during differentiation [31]; most retain H3K4me3 in their specific cell type and retain H3K27me3 in alternate cell fates [31,55]. Although fewer bivalent genes have been observed in differentiated cell types than in stem cells, it is unclear how bivalency resolves during differentiation of progenitor cells to their direct downstream lineages *in vivo*. Our analysis of hematopoietic stem and progenitor cells (HSPCs) and *in vitro* differentiated erythrocyte precursors (pRBCs), *in vivo* differentiated CD4+ T-cells, and CD19+ B-cells in the blood compartment indicated that many genes critical for HSPC differentiation are primed by bivalent modification and may also be bound by PolII in HSPCs prior to differentiation. E.g., the T-cell regulator *GATA3* and the B-cell master regulator *PAX5* were both bivalent in HSPCs; they became uniquely expressed and resolved to monovalently active in T and B-cells, respectively. PolII bound *PAX5* in HSPCs. Bivalency is not limited to promoters; we noted several enhancer-like regions showing the presence of both active and repressive marks in specific cell types (DATA NOT SHOWN). As suggested by previous publications, bivalency primes these critical genes for expression during differentiation [18,31]. Obviously, bivalent genes are heterogeneous in both histone modification enrichments and expression behavior. They can be separated into different groups based on the ratio between H3K4me3 and H3K27me3 enrichment, and the enormous variation in this ratio translates into important differences in gene expression behavior [18,56,57].

Curiously, *GATA3* remained bivalent in B-cells and *PAX5* remained bivalent in T-cells, indicating that these closely related lymphocyte types might possess some potential for plasticity between them. This is consistent with a previous report that bivalent modification of key regulators in T helper cells are linked to their plasticity [58]. Although *PAX5* is bivalent in both the progenitor HSPCs and T-cells, the chromatin remodeling factor BRG1 and PolII are associated with the promoter in HSPCs but not in T-cells, suggesting that changes in chromatin and expression potential have occurred after differentiation to T-cells. In contrast, both the *GATA3* and *PAX5* genes lost H3K4me3 in pRBCs, silencing them in this distantly related cell type, indicating little likelihood of direct transdifferentiation between these lymphoid and myeloid cell types. Indeed, conversion of *Pax5*-deleted B-cells into T-cells has been performed, albeit through de-differentiation into progenitor cell types [59]. Factors implicated in chromatin structure and modification, C/EBPα and C/EBPβ, can transdifferentiate B-cells into macrophage-like cells [60,61]. GATA3 itself was shown to mediate histone modifications at great distances from its binding sites in mouse [44]. Together, these results indicate substantial opportunities for epigenetic-mediated transdifferentiation of blood lineages, which is furthered by our analysis of incomplete silencing of TS factors in parallel cell types

Establishment of novel bivalency in partially differentiated progenitors is an underaddressed trait of development. Several genes that were not bivalent in HSPCs became bivalent downstream. Strikingly few genes had neither mark in HSPCs and subsequently become bivalent. Bivalency seems to thus be both established and resolved in a stepwise manner, as relatively few genes lose both active and repressive marks between differentiation stages (Figure 4B). For example, while H3K27me3 at the *HOX* loci changes drastically from ESC to HSPC and beyond (Additional file 1: Figure S3), most of the genes in these clusters retained H3K4me3 in the promoter regions (DATA NOT SHOWN). This may be explained as H3K27me3 targeting genes for selective repression initially, while H3K4me3 changes are secondary or occur on final commitment. In fact, we see that many more changes in H3K4me3 occur between HSPCs and the strongly committed pRBCs, while most other notable changes between cell types are H3K27me3-related. The stability of promoter H3K4me3 may be related to the presence of CpG islands within the promoter. This establishment of bivalency in non stem-like populations may require further attention to fully investigate its importance. We acknowledge that there are other silencing mechanisms such as H3K9me3 or DNA methylation in the cells, which may or may not overlap with the H3K27me3 pathway. More analyses of these different chromatin modifications are required for a more complete understanding of gene priming, repression and activation during differentiation of these cells.

ChIP-Seq is performed on a population of cells, and homogeneity of this population is crucial to analyses that describe subsets [16]. Although the cells used in this study were defined and purity-assessed by the cell surface markers, bivalent modification detection may have resulted from cellular heterogeneity [62]. We have previously shown [18] via sequential ChIP that both H3K4me3 and H3K27me3 can exist at the same promoter, but whether or not these marks exist on the same histone, or are functional in a single-nucleosome context is still unknown.

## Conclusion

In this study we identified tissue-specific genes in the human blood compartment and compared gene expression status with chromatin modification patterns. Based on the gene expression and chromatin modification patterns, we have predicted tens of thousands of potential core regulatory elements shared by all cell types and potential tissue-specific regulatory elements and show that a combination of both H3K4me1 and H2A.Z is a better predictor of enhancer activity than either alone. We have shown that bivalent chromatin modification in the well-characterized human hematopoietic system is not only resolved but also established during differentiation in a generally stepwise manner. While much of H3K4me3 enrichment is stable across cell types, H3K27me3 varies more frequently and grows to cover more of the genome during differentiation. These epigenomic data and identification of potential regulatory elements will be useful for further understanding the mechanisms governing decision making and differentiation of hematopoietic cell types.

## Methods

### Data sources

Data for CD34+/CD133+ hematopoietic stem and progenitor cells, CD36+ red blood cell precursors, and CD4+ T cells were downloaded from the NCBI Short Read Archive. A full summary of data sources is available as Additional file 1: Table 3. Counts of uniquely mapped reads in library, non-redundant reads, and reads falling in statistically enriched islands are Additional file 1: Table 4. New data can be downloaded via NCBI GEO using accession GSE39229.

Naïve B cells were purified from human blood using human Naïve B cell isolation kit II kits (Miltenyi, #130-091-150). The cells were digested with MNase to generate mainly mononucleosomes with minor fraction of dinucleosomes for histone modification mapping. For mapping enzyme target sites, the cells were crosslinked with formaldehyde treatment and chromatin fragmented to 200 to 500 bp by sonication. Chromatin from 5 × 10^6^ cells was used for each ChIP experiment.

### ChIP-Seq and analysis

ChIP-Seq was performed as previously described [16]. Illumina reads were mapped to the hg18 genome using Bowtie [63], allowing only one position per read (−m 1), and filtered to allow only one read per position. Y chromosome reads were disregarded.

We used MACS version 1.4.0 RC 2 [64] with input control libraries from corresponding cell types, a p-value threshold of 1 × 10^-8^, and –g hs to detect high-confidence Pol II-binding sites. Genes were considered Pol II-bound if they contained a peak between 5 kbp upstream of the TSS to 3 kbp downstream of the TES.

Regions enriched in Brg1 or modified or variant histones were detected using SICER version 1.03 [65]. The percent of hg18 uniquely mappable by 25 bp reads (68%) was retrieved from [66]. We used a window size of 200 for Brg1, H2A.Z, H3K4me1, and H3K4me3. Window sizes for H3K27me1 and H3K27me3 were predicted using an unpublished version of SICER [IN PREPARATION vs In Preparation]. We used gap sizes of 0 windows for H3K27me1 and H3K27me3, 1 window for H3K4me1 and H3K4me3, 2 windows for H2A.Z, and 3 windows for Brg1. Fragment size was 150, and the FDR cutoff for statistical enrichment was 1 × 10^-5^. Due to the potentially large size of windows, we nibbled the edges of outermost windows of each island to maximize the difference in read density between the remaining portion and the removed portion of the outermost windows. This feature will be implemented in the next version of SICER [IN PREPARATION vs In Preparation]. UCSC browser tracks were created using 200 bp window sizes, a +/− 75 bp shift by strand, and were normalized to the 10^7^ reads in the library.

Reads used for alignments with respect to TSSs and enhancers were required to come from statistically enriched islands. For enhancers, reads were shifted +/− 75 strand-dependent bps, sorted by their starts into 50 equally sized bins, counted, and normalized by bin size, TSS number, and sequencing read count. For TSS profiles, reads were aligned relative to RefSeq TSSs separated by strand, counted in 10 bp bins, inverted for negative strand genes, summed, smoothed over 4 windows on either side, and normalized by sequencing read count. Both sets of profiles were plotted using GNUPLOT [67]. We used MeV [68,69] to display heatmaps in Figure 3 A, C, and D, without correction of zeroes.

Figure 3B area-proportional Venn diagrams were created using the VennDiagram package in R [70]. CpG islands were downloaded from the UCSC Genome Browser [71]

Figure 3F shows a heatmap of H3K27me3 reads. Islands of H3K27me3 from all five cell types were united, and fragmented equally into ≤ 2 kbp fragments. Read counts of H3K27me3 in these fragments were normalized by sequencing library size, clustered using k means (k = 20) clustering, and displayed in a heatmap using R, sorted by cluster sum of H3K27me3 reads [70].

Promoters were considered to be bivalent if they had a region statistically enriched in both H3K4me3 and H3K27me3 within 500 bp of the TSS. Enhancers were considered bivalent if they had any H3K27me3 enrichment in their locus. Type-specific transcription factors bivalently prepared in HSPCs were predicted using GeneCards version 3 [72] to extract Gene Ontology terms [73], and required to have the term “transcription factor.”

We predicted enhancers by taking the union of H2A.Z- and H3K4me1-enriched islands using SICER. Enhancers, and H3K27me3 regions in Figure 3F were associated with transcripts if they were located between 20 kbp upstream of the TSS and 0.5 kbp upstream of the TSS, or between TES and 20 kbp downstream of the TES. P-values in Figure 7A were calculated using a two-tailed Kolmogorof-Smirnoff test in R version 2.6.2 [70]. The highest 5% of RPKM values were removed from distribution analysis in Figure 7B.

### RNA-Seq and analysis

RNA-Seq was performed as previously described [8]. Illumina reads were aligned to the hg18 genome using TopHat [74] with standard parameters. Mapped reads were then converted to BED format using SAMtools [75]. UCSC browser tracks [71] were created from BED reads using no shift and a window size of 20bp. We calculated RPKM values for all RefSeq transcripts not on the Y chromosome using a previously described method [8]. Y chromsome reads were disregarded as some subjects were female. Pairwise differential expression was calculated using EdgeR [76] with log(fold-change) ≥ 5 and FDR ≤ 1 × 10^-5^ thresholds. We found type-specific genes by taking the intersection of the three lists of significantly more highly expressed genes from the three pairwise comparisons per cell type. KEGG pathway analysis was performed using DAVID [77] KEGG pathway enrichment with standard settings.

## Competing interests

The authors declare that they have no competing interests.

## Authors’ contributions

KZ conceived the project. KC and QT performed the experiments and BJA analyzed the data. BJA and KZ wrote the paper. All authors read and approved the final manuscript.

## Supplementary Material

Additional file 1 **Methods. Figure S1A-S1D**: Expression of type-specific genes in four cell types – related to **Figure **1. **Figure S2A-S2D.** Type-specific genes are enriched in functional pathways – related to **Figure **1. **Figure S3A-S3B.** Enrichment of H3K27me3 at *HOXA* and *HOXB* loci – related to Figure 3. **Figure S4.** Bivalency of promoters across multiple cell types with examples – related to Figure 3. **Figure S5A-B.** Density of chromatin proteins at *Gata3* and *Pax5* genes – related to Figure 4. **Figure S6.** Resolution and preparation of transcription factor genes bivalent in HSPC – related to Figure 3. **Table S1.** Excel document of lists of type-specific genes. **Table S2.** Known enhancers and their enrichments in H3K4me1 and/or H2A.Z in four cell types. **Table S3.** Files used in analysis and their sources. **Table S4.** Mapped sequencing reads, unique reads, and unique reads in enriched islands. **Discussion of Figure7B.** Click here for file

## References

[B1] Till JE McCulloch EA A direct measurement of the radiation sensitivity of normal mouse bone marrow cells Radiat Res 1961 14 213 222 10.2307/3570892 13776896

[B2] Freyssinier JM Lecoq-Lafon C Amsellem S Picard F Ducrocq R Mayeux P Lacombe C Fichelson S Purification, amplification and characterization of a population of human erythroid progenitors Br J Haematol 1999 106 4 912 922 10.1046/j.1365-2141.1999.01639.x 10519992

[B3] Giarratana MC Kobari L Lapillonne H Chalmers D Kiger L Cynober T Marden MC Wajcman H Douay L Ex vivo generation of fully mature human red blood cells from hematopoietic stem cells Nat Biotechnol 2005 23 1 69 74 10.1038/nbt1047 15619619

[B4] Hattangadi SM Wong P Zhang L Flygare J Lodish HF From stem cell to red cell: regulation of erythropoiesis at multiple levels by multiple proteins, RNAs, and chromatin modifications Blood 2011 118 24 6258 6268 10.1182/blood-2011-07-356006 21998215 PMC3236116

[B5] Kawamoto H Ikawa T Masuda K Wada H Katsura Y A map for lineage restriction of progenitors during hematopoiesis: the essence of the myeloid-based model Immunol Rev 2010 238 1 23 36 10.1111/j.1600-065X.2010.00959.x 20969582

[B6] Kerenyi MA Orkin SH Networking erythropoiesis J Exp Med 2010 207 12 2537 2541 10.1084/jem.20102260 21098097 PMC2989762

[B7] Warren LA Rothenberg EV Regulatory coding of lymphoid lineage choice by hematopoietic transcription factors Curr Opin Immunol 2003 15 2 166 175 10.1016/S0952-7915(03)00011-6 12633666

[B8] Hu G Schones DE Cui K Ybarra R Northrup D Tang Q Gattinoni L Restifo NP Huang S Zhao K Regulation of nucleosome landscape and transcription factor targeting at tissue-specific enhancers by BRG1 Genome Res 2011 21 10 1650 1658 10.1101/gr.121145.111 21795385 PMC3202282

[B9] Kidder BL Palmer S Knott JG SWI/SNF-Brg1 regulates self-renewal and occupies core pluripotency-related genes in embryonic stem cells Stem Cells 2009 27 2 317 328 10.1634/stemcells.2008-0710 19056910

[B10] Kim SI Bresnick EH Bultman SJ BRG1 directly regulates nucleosome structure and chromatin looping of the alpha globin locus to activate transcription Nucleic Acids Res 2009 37 18 6019 6027 10.1093/nar/gkp677 19696073 PMC2764439

[B11] Kim SI Bultman SJ Kiefer CM Dean A Bresnick EH BRG1 requirement for long-range interaction of a locus control region with a downstream promoter Proc Natl Acad Sci U S A 2009 106 7 2259 2264 10.1073/pnas.0806420106 19171905 PMC2650142

[B12] Ho L Jothi R Ronan JL Cui K Zhao K Crabtree GR An embryonic stem cell chromatin remodeling complex, esBAF, is an essential component of the core pluripotency transcriptional network Proc Natl Acad Sci U S A 2009 106 13 5187 5191 10.1073/pnas.0812888106 19279218 PMC2654397

[B13] Eissenberg JC Shilatifard A Histone H3 lysine 4 (H3K4) methylation in development and differentiation Dev Biol 2010 339 2 240 249 10.1016/j.ydbio.2009.08.017 19703438 PMC3711867

[B14] Ruthenburg AJ Allis CD Wysocka J Methylation of lysine 4 on histone H3: intricacy of writing and reading a single epigenetic mark Mol Cell 2007 25 1 15 30 10.1016/j.molcel.2006.12.014 17218268

[B15] Sims RJ 3rd Reinberg D Histone H3 Lys 4 methylation: caught in a bind? Genes Dev 2006 20 20 2779 2786 10.1101/gad.1468206 17043307

[B16] Barski A Cuddapah S Cui K Roh TY Schones DE Wang Z Wei G Chepelev I Zhao K High-resolution profiling of histone methylations in the human genome Cell 2007 129 4 823 837 10.1016/j.cell.2007.05.009 17512414

[B17] Kim TH Barrera LO Zheng M Qu C Singer MA Richmond TA Wu Y Green RD Ren B A high-resolution map of active promoters in the human genome Nature 2005 436 7052 876 880 10.1038/nature03877 15988478 PMC1895599

[B18] Roh TY Cuddapah S Cui K Zhao K The genomic landscape of histone modifications in human T cells Proc Natl Acad Sci U S A 2006 103 43 15782 15787 10.1073/pnas.0607617103 17043231 PMC1613230

[B19] Bernstein BE Kamal M Lindblad-Toh K Bekiranov S Bailey DK Huebert DJ McMahon S Karlsson EK Kulbokas EJ 3rd Gingeras TR Genomic maps and comparative analysis of histone modifications in human and mouse Cell 2005 120 2 169 181 10.1016/j.cell.2005.01.001 15680324

[B20] Mohn F Weber M Rebhan M Roloff TC Richter J Stadler MB Bibel M Schubeler D Lineage-specific polycomb targets and de novo DNA methylation define restriction and potential of neuronal progenitors Mol Cell 2008 30 6 755 766 10.1016/j.molcel.2008.05.007 18514006

[B21] Boyer LA Plath K Zeitlinger J Brambrink T Medeiros LA Lee TI Levine SS Wernig M Tajonar A Ray MK Polycomb complexes repress developmental regulators in murine embryonic stem cells Nature 2006 441 7091 349 353 10.1038/nature04733 16625203

[B22] Simon JA Kingston RE Mechanisms of polycomb gene silencing: knowns and unknowns Nat Rev Mol Cell Biol 2009 10 10 697 708 19738629 10.1038/nrm2763

[B23] Hansen KH Bracken AP Pasini D Dietrich N Gehani SS Monrad A Rappsilber J Lerdrup M Helin K A model for transmission of the H3K27me3 epigenetic mark Nat Cell Biol 2008 10 11 1291 1300 10.1038/ncb1787 18931660

[B24] Shilatifard A Chromatin modifications by methylation and ubiquitination: implications in the regulation of gene expression Annu Rev Biochem 2006 75 243 269 10.1146/annurev.biochem.75.103004.142422 16756492

[B25] Cao R Wang L Wang H Xia L Erdjument-Bromage H Tempst P Jones RS Zhang Y Role of histone H3 lysine 27 methylation in Polycomb-group silencing Science 2002 298 5595 1039 1043 10.1126/science.1076997 12351676

[B26] Yang X Noushmehr H Han H Andreu-Vieyra C Liang G Jones PA Gene reactivation by 5-aza-2’-deoxycytidine-induced demethylation requires SRCAP-mediated H2A.Z insertion to establish nucleosome depleted regions PLoS Genet 2012 8 3 e1002604 10.1371/journal.pgen.1002604 22479200 PMC3315468

[B27] Jin C Zang C Wei G Cui K Peng W Zhao K Felsenfeld G H3.3/H2A.Z double variant-containing nucleosomes mark ‘nucleosome-free regions’ of active promoters and other regulatory regions Nat Genet 2009 41 8 941 945 10.1038/ng.409 19633671 PMC3125718

[B28] Guillemette B Bataille AR Gevry N Adam M Blanchette M Robert F Gaudreau L Variant histone H2A.Z is globally localized to the promoters of inactive yeast genes and regulates nucleosome positioning PLoS Biol 2005 3 12 e384 10.1371/journal.pbio.0030384 16248679 PMC1275524

[B29] Ernst J Kheradpour P Mikkelsen TS Shoresh N Ward LD Epstein CB Zhang X Wang L Issner R Coyne M Mapping and analysis of chromatin state dynamics in nine human cell types Nature 2011 473 7345 43 49 10.1038/nature09906 21441907 PMC3088773

[B30] Ram O Goren A Amit I Shoresh N Yosef N Ernst J Kellis M Gymrek M Issner R Coyne M Combinatorial patterning of chromatin regulators uncovered by genome-wide location analysis in human cells Cell 2011 147 7 1628 1639 10.1016/j.cell.2011.09.057 22196736 PMC3312319

[B31] Bernstein BE Mikkelsen TS Xie X Kamal M Huebert DJ Cuff J Fry B Meissner A Wernig M Plath K A bivalent chromatin structure marks key developmental genes in embryonic stem cells Cell 2006 125 2 315 326 10.1016/j.cell.2006.02.041 16630819

[B32] Cui K Zang C Roh TY Schones DE Childs RW Peng W Zhao K Chromatin signatures in multipotent human hematopoietic stem cells indicate the fate of bivalent genes during differentiation Cell Stem Cell 2009 4 1 80 93 10.1016/j.stem.2008.11.011 19128795 PMC2785912

[B33] Mikkelsen TS Ku M Jaffe DB Issac B Lieberman E Giannoukos G Alvarez P Brockman W Kim TK Koche RP Genome-wide maps of chromatin state in pluripotent and lineage-committed cells Nature 2007 448 7153 553 560 10.1038/nature06008 17603471 PMC2921165

[B34] Sanz LA Chamberlain S Sabourin JC Henckel A Magnuson T Hugnot JP Feil R Arnaud P A mono-allelic bivalent chromatin domain controls tissue-specific imprinting at Grb10 EMBO J 2008 27 19 2523 2532 10.1038/emboj.2008.142 18650936 PMC2567399

[B35] Azuara V Perry P Sauer S Spivakov M Jorgensen HF John RM Gouti M Casanova M Warnes G Merkenschlager M Chromatin signatures of pluripotent cell lines Nat Cell Biol 2006 8 5 532 538 10.1038/ncb1403 16570078

[B36] Pan G Tian S Nie J Yang C Ruotti V Wei H Jonsdottir GA Stewart R Thomson JA Whole-genome analysis of histone H3 lysine 4 and lysine 27 methylation in human embryonic stem cells Cell Stem Cell 2007 1 3 299 312 10.1016/j.stem.2007.08.003 18371364

[B37] Zhao XD Han X Chew JL Liu J Chiu KP Choo A Orlov YL Sung WK Shahab A Kuznetsov VA Whole-genome mapping of histone H3 Lys4 and 27 trimethylations reveals distinct genomic compartments in human embryonic stem cells Cell Stem Cell 2007 1 3 286 298 10.1016/j.stem.2007.08.004 18371363

[B38] Chepelev I Wei G Tang Q Zhao K Detection of single nucleotide variations in expressed exons of the human genome using RNA-Seq Nucleic Acids Res 2009 37 16 e106 10.1093/nar/gkp507 19528076 PMC2760790

[B39] Lister R Pelizzola M Kida YS Hawkins RD Nery JR Hon G Antosiewicz-Bourget J O’Malley R Castanon R Klugman S Hotspots of aberrant epigenomic reprogramming in human induced pluripotent stem cells Nature 2011 471 7336 68 73 10.1038/nature09798 21289626 PMC3100360

[B40] Gardiner-Garden M Frommer M CpG islands in vertebrate genomes J Mol Biol 1987 196 2 261 282 10.1016/0022-2836(87)90689-9 3656447

[B41] Hawkins RD Hon GC Lee LK Ngo Q Lister R Pelizzola M Edsall LE Kuan S Luu Y Klugman S Distinct epigenomic landscapes of pluripotent and lineage-committed human cells Cell Stem Cell 2010 6 5 479 491 10.1016/j.stem.2010.03.018 20452322 PMC2867844

[B42] Zhu J Yamane H Paul WE Differentiation of effector CD4 T cell populations (*) Annu Rev Immunol 2010 28 445 489 10.1146/annurev-immunol-030409-101212 20192806 PMC3502616

[B43] Wu JQ Seay M Schulz VP Hariharan M Tuck D Lian J Du J Shi M Ye Z Gerstein M Tcf7 is an important regulator of the switch of self-renewal and differentiation in a multipotential hematopoietic cell line PLoS Genet 2012 8 3 e1002565 10.1371/journal.pgen.1002565 22412390 PMC3297581

[B44] Wei G Abraham BJ Yagi R Jothi R Cui K Sharma S Narlikar L Northrup DL Tang Q Paul WE Genome-wide analyses of transcription factor GATA3-mediated gene regulation in distinct T cell types Immunity 2009 35 2 299 311 21867929 10.1016/j.immuni.2011.08.007 PMC3169184

[B45] Heintzman ND Hon GC Hawkins RD Kheradpour P Stark A Harp LF Ye Z Lee LK Stuart RK Ching CW Histone modifications at human enhancers reflect global cell-type-specific gene expression Nature 2009 459 7243 108 112 10.1038/nature07829 19295514 PMC2910248

[B46] Rada-Iglesias A Bajpai R Swigut T Brugmann SA Flynn RA Wysocka J A unique chromatin signature uncovers early developmental enhancers in humans Nature 2011 470 7333 279 283 10.1038/nature09692 21160473 PMC4445674

[B47] Roh TY Cuddapah S Zhao K Active chromatin domains are defined by acetylation islands revealed by genome-wide mapping Genes Dev 2005 19 5 542 552 10.1101/gad.1272505 15706033 PMC551575

[B48] Roh TY Wei G Farrell CM Zhao K Genome-wide prediction of conserved and nonconserved enhancers by histone acetylation patterns Genome Res 2007 17 1 74 81 17135569 10.1101/gr.5767907 PMC1716270

[B49] Creyghton MP Cheng AW Welstead GG Kooistra T Carey BW Steine EJ Hanna J Lodato MA Frampton GM Sharp PA Histone H3K27ac separates active from poised enhancers and predicts developmental state Proc Natl Acad Sci U S A 2010 107 50 21931 21936 10.1073/pnas.1016071107 21106759 PMC3003124

[B50] Heintzman ND Stuart RK Hon G Fu Y Ching CW Hawkins RD Barrera LO Van Calcar S Qu C Ching KA Distinct and predictive chromatin signatures of transcriptional promoters and enhancers in the human genome Nat Genet 2007 39 3 311 318 10.1038/ng1966 17277777

[B51] Wang Z Zang C Rosenfeld JA Schones DE Barski A Cuddapah S Cui K Roh TY Peng W Zhang MQ Combinatorial patterns of histone acetylations and methylations in the human genome Nat Genet 2008 40 7 897 903 10.1038/ng.154 18552846 PMC2769248

[B52] Steiner LA Schulz VP Maksimova Y Wong C Gallagher PG Patterns of histone H3 lysine 27 monomethylation and erythroid cell type-specific gene expression J Biol Chem 2011 286 45 39457 39465 10.1074/jbc.M111.243006 21937433 PMC3234769

[B53] Pekowska A Benoukraf T Zacarias-Cabeza J Belhocine M Koch F Holota H Imbert J Andrau JC Ferrier P Spicuglia S H3K4 tri-methylation provides an epigenetic signature of active enhancers EMBO J 2011 30 20 4198 4210 10.1038/emboj.2011.295 21847099 PMC3199384

[B54] Ku M Koche RP Rheinbay E Mendenhall EM Endoh M Mikkelsen TS Presser A Nusbaum C Xie X Chi AS Genomewide analysis of PRC1 and PRC2 occupancy identifies two classes of bivalent domains PLoS Genet 2008 4 10 e1000242 10.1371/journal.pgen.1000242 18974828 PMC2567431

[B55] van Arensbergen J Garcia-Hurtado J Moran I Maestro MA Xu X Van de Casteele M Skoudy AL Palassini M Heimberg H Ferrer J Derepression of polycomb targets during pancreatic organogenesis allows insulin-producing beta-cells to adopt a neural gene activity program Genome Res 2010 20 6 722 732 10.1101/gr.101709.109 20395405 PMC2877569

[B56] De Gobbi M Garrick D Lynch M Vernimmen D Hughes JR Goardon N Luc S Lower KM Sloane-Stanley JA Pina C Generation of bivalent chromatin domains during cell fate decisions Epigenetics Chromatin 2011 4 1 9 10.1186/1756-8935-4-9 21645363 PMC3131236

[B57] Gibson JD Jakuba CM Boucher N Holbrook KA Carter MG Nelson CE Single-cell transcript analysis of human embryonic stem cells Integr Biol (Camb) 2009 1 8–9 540 551 20023769 10.1039/b908276j

[B58] Bending D Newland S Krejci A Phillips JM Bray S Cooke A Epigenetic changes at Il12rb2 and Tbx21 in relation to plasticity behavior of Th17 cells J Immunol 2011 186 6 3373 3382 10.4049/jimmunol.1003216 21307296

[B59] Cobaleda C Jochum W Busslinger M Conversion of mature B cells into T cells by dedifferentiation to uncommitted progenitors Nature 2007 449 7161 473 477 10.1038/nature06159 17851532

[B60] Xie H Ye M Feng R Graf T Stepwise reprogramming of B cells into macrophages Cell 2004 117 5 663 676 10.1016/S0092-8674(04)00419-2 15163413

[B61] Kovacs KA Steinmann M Magistretti PJ Halfon O Cardinaux JR CCAAT/enhancer-binding protein family members recruit the coactivator CREB-binding protein and trigger its phosphorylation J Biol Chem 2003 278 38 36959 36965 10.1074/jbc.M303147200 12857754

[B62] Wang Z Schones DE Zhao K Characterization of human epigenomes Curr Opin Genet Dev 2009 19 2 127 134 10.1016/j.gde.2009.02.001 19299119 PMC2699568

[B63] Langmead B Trapnell C Pop M Salzberg SL Ultrafast and memory-efficient alignment of short DNA sequences to the human genome Genome Biol 2009 10 3 R25 10.1186/gb-2009-10-3-r25 19261174 PMC2690996

[B64] Zhang Y Liu T Meyer CA Eeckhoute J Johnson DS Bernstein BE Nusbaum C Myers RM Brown M Li W Model-based analysis of ChIP-Seq (MACS) Genome Biol 2008 9 9 R137 10.1186/gb-2008-9-9-r137 18798982 PMC2592715

[B65] Zang C Schones DE Zeng C Cui K Zhao K Peng W A clustering approach for identification of enriched domains from histone modification ChIP-Seq data Bioinformatics 2009 25 15 1952 1958 10.1093/bioinformatics/btp340 19505939 PMC2732366

[B66] Koehler R Issac H Cloonan N Grimmond SM The uniqueome: a mappability resource for short-tag sequencing Bioinformatics 2010 27 2 272 274 21075741 10.1093/bioinformatics/btq640 PMC3018812

[B67] Williams T Kelley C Gnuplot 4.2: an interactive plotting program 2011

[B68] Saeed AI Bhagabati NK Braisted JC Liang W Sharov V Howe EA Li J Thiagarajan M White JA Quackenbush J TM4 microarray software suite Methods Enzymol 2006 411 134 193 16939790 10.1016/S0076-6879(06)11009-5

[B69] Saeed AI Sharov V White J Li J Liang W Bhagabati N Braisted J Klapa M Currier T Thiagarajan M TM4: a free, open-source system for microarray data management and analysis Biotechniques 2003 34 2 374 378 12613259 10.2144/03342mt01

[B70] R Development Core Team R: A language and environment for statistical computing 2010 Vienna, Austria: R Foundation for Statistical Computing

[B71] Kent WJ Sugnet CW Furey TS Roskin KM Pringle TH Zahler AM Haussler D The human genome browser at UCSC Genome Res 2002 12 6 996 1006 12045153 10.1101/gr.229102 PMC186604

[B72] Safran M Dalah I Alexander J Rosen N Iny Stein T Shmoish M Nativ N Bahir I Doniger T Krug H GeneCards version 3: the human gene integrator Database (Oxford) 2010 2010 baq020 10.1093/database/baq020 20689021 PMC2938269

[B73] Ashburner M Ball CA Blake JA Botstein D Butler H Cherry JM Davis AP Dolinski K Dwight SS Eppig JT Gene ontology: tool for the unification of biology. The Gene Ontology Consortium Nat Genet 2000 25 1 25 29 10.1038/75556 10802651 PMC3037419

[B74] Trapnell C Pachter L Salzberg SL TopHat: discovering splice junctions with RNA-Seq Bioinformatics 2009 25 9 1105 1111 10.1093/bioinformatics/btp120 19289445 PMC2672628

[B75] Li H Handsaker B Wysoker A Fennell T Ruan J Homer N Marth G Abecasis G Durbin R The sequence alignment/Map format and SAMtools Bioinformatics 2009 25 16 2078 2079 10.1093/bioinformatics/btp352 19505943 PMC2723002

[B76] Robinson MD McCarthy DJ Smyth GK edgeR: a bioconductor package for differential expression analysis of digital gene expression data Bioinformatics 2010 26 1 139 140 10.1093/bioinformatics/btp616 19910308 PMC2796818

[B77] Dennis G Jr Sherman BT Hosack DA Yang J Gao W Lane HC Lempicki RA DAVID: database for annotation, visualization, and integrated discovery Genome Biol 2003 4 5 P3 10.1186/gb-2003-4-5-p3 12734009 PMC193660

