# Supplementary material for: Dynamic regulation of epigenomic landscapes during hematopoiesis
Source: BMC Genomics. 2013 Mar 19;14:193. doi: 10.1186/1471-2164-14-193 (PMC3636055; doi:10.1186/1471-2164-14-193)
Supplement: Additional file 1 — Methods. Figure S1A-S1D: Expression of type-specific genes in four cell types – related to Figure 1. Figure S2A-S2D. Type-specific genes are enriched in functional pathways – related to Figure 1. Figure S3A-S3B. Enrichment of H3K27me3 at HOXA and HOXB loci – related to Figure 3. Figure S4. Bivalency of promoters across multiple cell types with examples – related to Figure 3. Figure S5A-B. Density of chromatin proteins at Gata3 and Pax5 genes – related to Figure 4. Figure S6. Resolution and preparation of transcription factor genes bivalent in HSPC – related to Figure 3. Table S1. Excel document of lists of type-specific genes. Table S2. Known enhancers and their enrichments in H3K4me1 and/or H2A.Z in four cell types. Table S3. Files used in analysis and their sources. Table S4. Mapped sequencing reads, unique reads, and unique reads in enriched islands. Discussion of Figure7B. [file 1471-2164-14-193-S1.doc]

**Supplementary Information**

# Dynamic Regulation of Epigenomic Landscapes During Hematopoiesis

Brian J. Abraham, Kairong Cui, Qingsong Tang, Keji Zhao

**Inventory of Supplementary Information**

1. **Supplementary Methods**
2. **Supplementary Figures S1-S4**

**Supplementary Figure S1A-S1D:** Expression of type-specific genes in four cell types – related to Figure 1.

**Supplementary Figure S2A-S2D:** Type-specific genes are enriched in functional pathways – related to Figure 1.

**Supplementary Figure S3A-S3B:** Enrichment of H3K27me3 at *HOXA* and *HOXB* loci – related to Figure 3.

**Supplementary Figure S4:** Bivalency of promoters across multiple cell types with examples – related to Figure 3.

**Supplementary Figure S5A-B:** Density of chromatin proteins at *Gata3* and *Pax5* genes – related to Figure 4

**Supplementary Figure S6:** Resolution and preparation of transcription factor genes bivalent in HSPC – related to Figure 3.

1. **Supplementary Tables S1-S4**

**Supplementary Table S1:** Excel document of lists of type-specific genes

**Supplementary Table S2:** Known enhancers and their enrichments in H3K4me1 and/or H2A.Z in four cell types

**Supplementary Table S3:** Files used in analysis and their sources

**Supplementary Table S4:** Mapped sequencing reads, unique reads, and unique reads in enriched islands

1. **Discussion of Figure 7B**
2. **Supplementary Methods:**

*RNA-Seq analysis:*

Illumina reads were aligned to the hg18 genome using TopHat[^1^](#_ENREF_1) with standard parameters. Mapped reads were then converted to BED format using SAMtools.[^2^](#_ENREF_2) [_ENREF_45](#_ENREF_45)We calculated RPKM (reads per kilobase of exon per million library reads) values for all RefSeq transcripts not on the Y chromosome using a previously described method.[^3^](#_ENREF_3) Y chromosome reads were disregarded as some subjects were female. Pairwise differential expression was calculated using EdgeR[^4^](#_ENREF_4) with “logFC” ≥ 5 and FDR ≤ 1x10^-5^ thresholds. Type-specific genes were defined as the intersection of the three lists of significantly more highly expressed genes from the three pairwise comparisons per cell type.

*UCSC browser tracks:*

UCSC browser tracks[^5^](#_ENREF_5) were created from BED reads using no shift and a window size of 20bp.

UCSC browser tracks were created using 200 bp window sizes, a +/- 75 bp shift by strand, and were normalized to the 10^7^ reads in the library.

*Read density alignment plots:*

Reads used for alignments with respect to TSSs and enhancers were required to come from statistically enriched islands. For enhancers, reads were shifted +/- 75 strand-dependent bps, sorted by their starts into 50 equally sized bins, counted, and normalized by bin size, TSS number, and sequencing read count. For TSS profiles, reads were aligned relative to RefSeq TSSs separated by strand, counted in 10 bp bins, inverted for negative strand genes, summed, smoothed over 4 windows on either side, and normalized by sequencing read count. Both sets of profiles were plotted using GNUPLOT.[^6^](#_ENREF_6) We used MeV to display heatmaps in Figure 3 A, C, and D, without correction of zeroes.

*Figure generation software:*

Figure 3B area-proportional Venn diagrams were created using the VennDiagram package in R.[^9^](#_ENREF_9) CpG islands were downloaded from the UCSC Genome Browser[^5^](#_ENREF_5)

Figure 3G shows a heatmap of H3K27me3 reads. Islands of H3K27me3 from all five cell types were united, and fragmented equally into ≤ 2 kbp fragments. Read counts of H3K27me3 in these fragments were normalized by sequencing library size, clustered using k means (k=20) clustering, and displayed in a heatmap using R, sorted by cluster sum of H3K27me3 reads.[^9^](#_ENREF_9)

*SICER analysis:*

The percent of hg18 uniquely mappable by 25 bp reads (68%) was retrieved from Uniqueome.[^10^](#_ENREF_10) We used a window size of 200 for Brg1, H2A.Z, H3K4me1, and H3K4me3. Window sizes for H3K27me1 and H3K27me3 were predicted using an unpublished version of SICER [In preparation].[^11^](#_ENREF_11) Briefly, the window size is determined by using an optimization approach developed for choosing bin size in histograms.[^12^](#_ENREF_12) For histone modifcations that form diffusive extended domains, such as H3K27me, the window size can be larger than the actual enriched region. To define the boundaries of ChIP-enriched regions with better resolution, the boundary windows are trimmed to maximize difference in enrichment density at the boundaries.

Based on these predictions, we used gap sizes of 0 windows for H3K27me1 and H3K27me3, 1 window for H3K4me1 and H3K4me3, 2 windows for H2A.Z, and 3 windows for Brg1. Fragment size was 150, and the FDR cutoff for statistical enrichment was 1x10^-5^. Due to the potentially large size of windows, we nibbled the edges of outermost windows of each island to maximize the difference in read density between the remaining portion and the removed portion of the outermost windows. This feature will be implemented in the next version of SICER [IN PREPARATION].

**
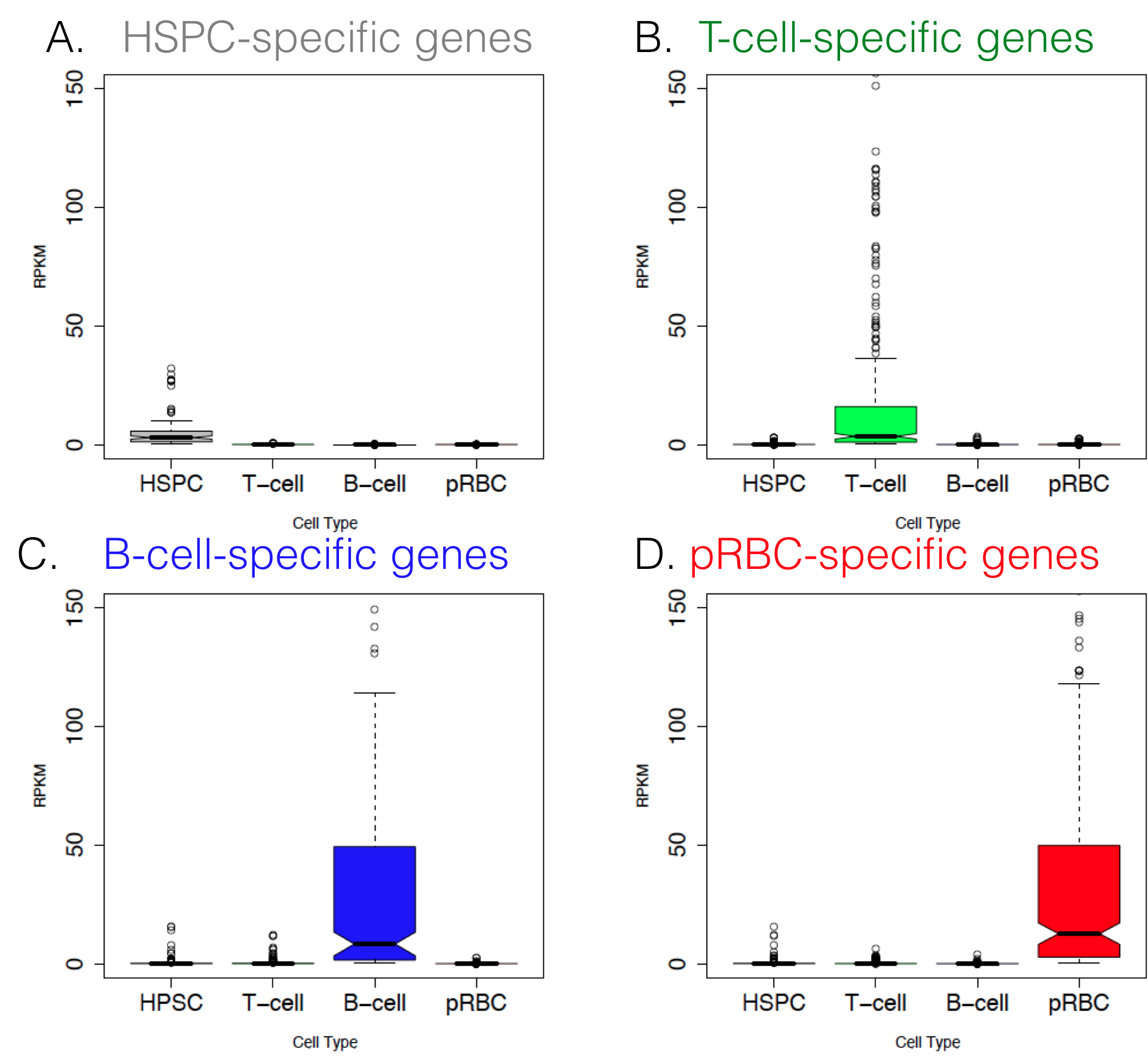
**

**Supplementary Figure S1A-S1D: Expression of type-specific genes in four cell types.** – related to Figure 1. RPKMs for genes calculated as specifically expressed in HSPC (A), T-cell (B), B-cell (C), and pRBC (D) are shown. Y ranges were limited to RPKM of 150 to better show relationships of lowly-expressed genes. All pair-wise comparisons between RPKM distribution of type-specific genes in their cell type and others have p-values < 2.2x10^-16^ by the Kolmogorov-Smirnov test.

**
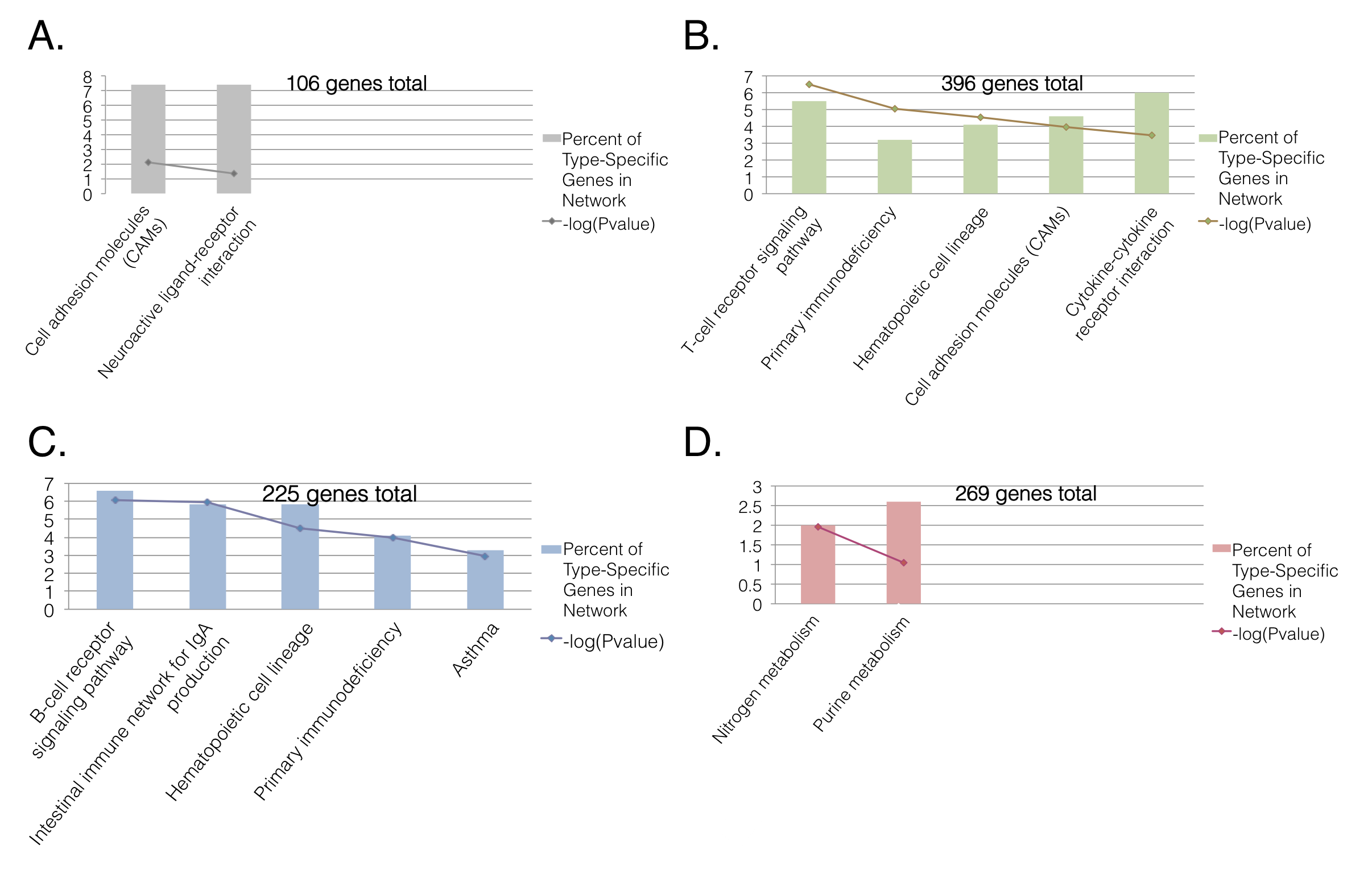
**

**Supplementary Figure S2A-S2D: Type-specific genes are enriched in functional pathways** – related to Figure 1. KEGG pathway analysis was performed using DAVID[^13^](#_ENREF_13) KEGG pathway enrichment with standard settings.

**A:** HSPC-specific genes are enriched in cell adhesion molecules and neuroactive ligand-receptor interaction KEGG pathways.

**B:** T cell-specific genes are enriched in KEGG pathways related to T cell function.

**C:** B cell-specific genes are enriched in KEGG pathways related to B cell function.

**D:** pRBC-specific genes are enriched in KEGG pathways for nitrogen and purine metabolism.

**
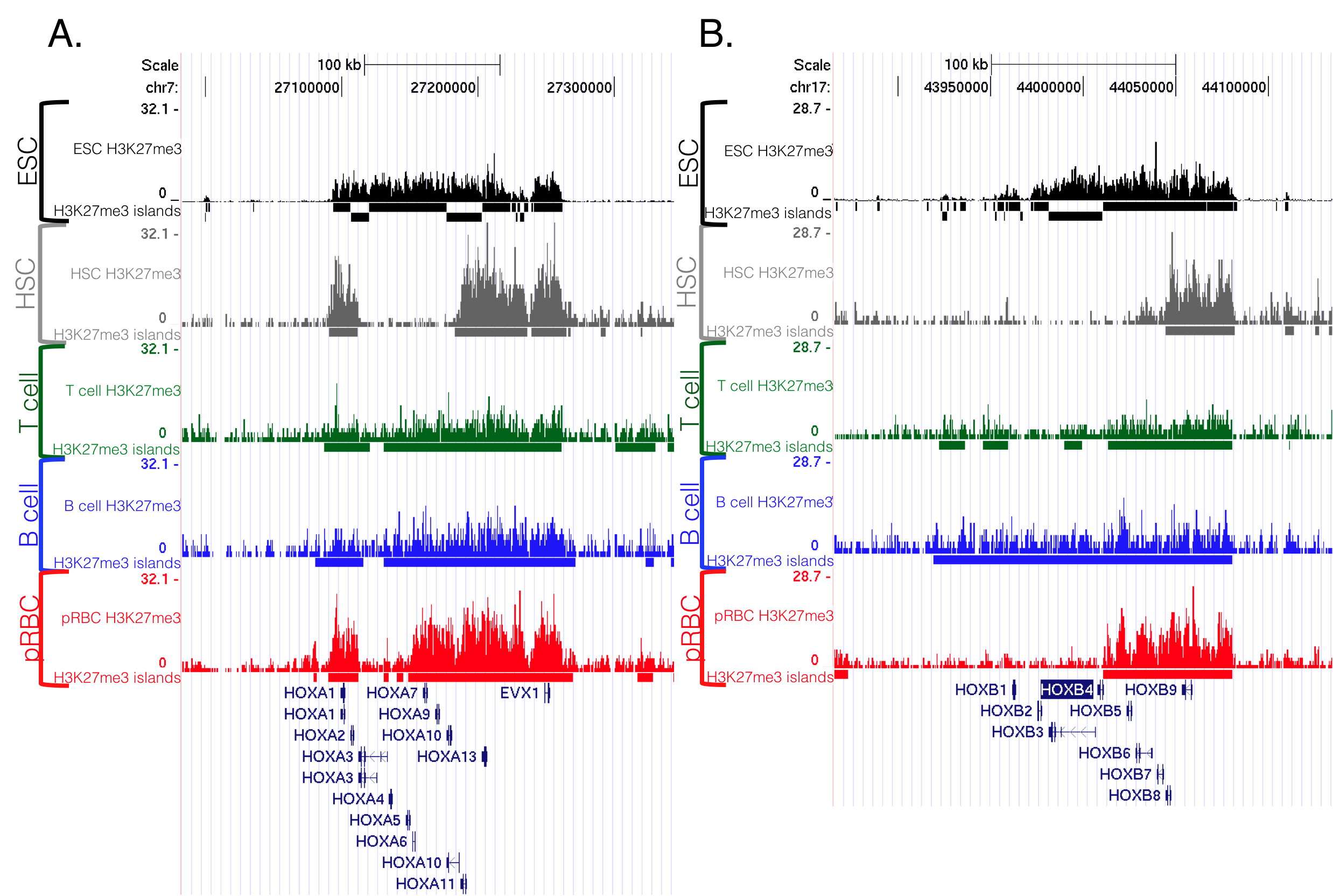

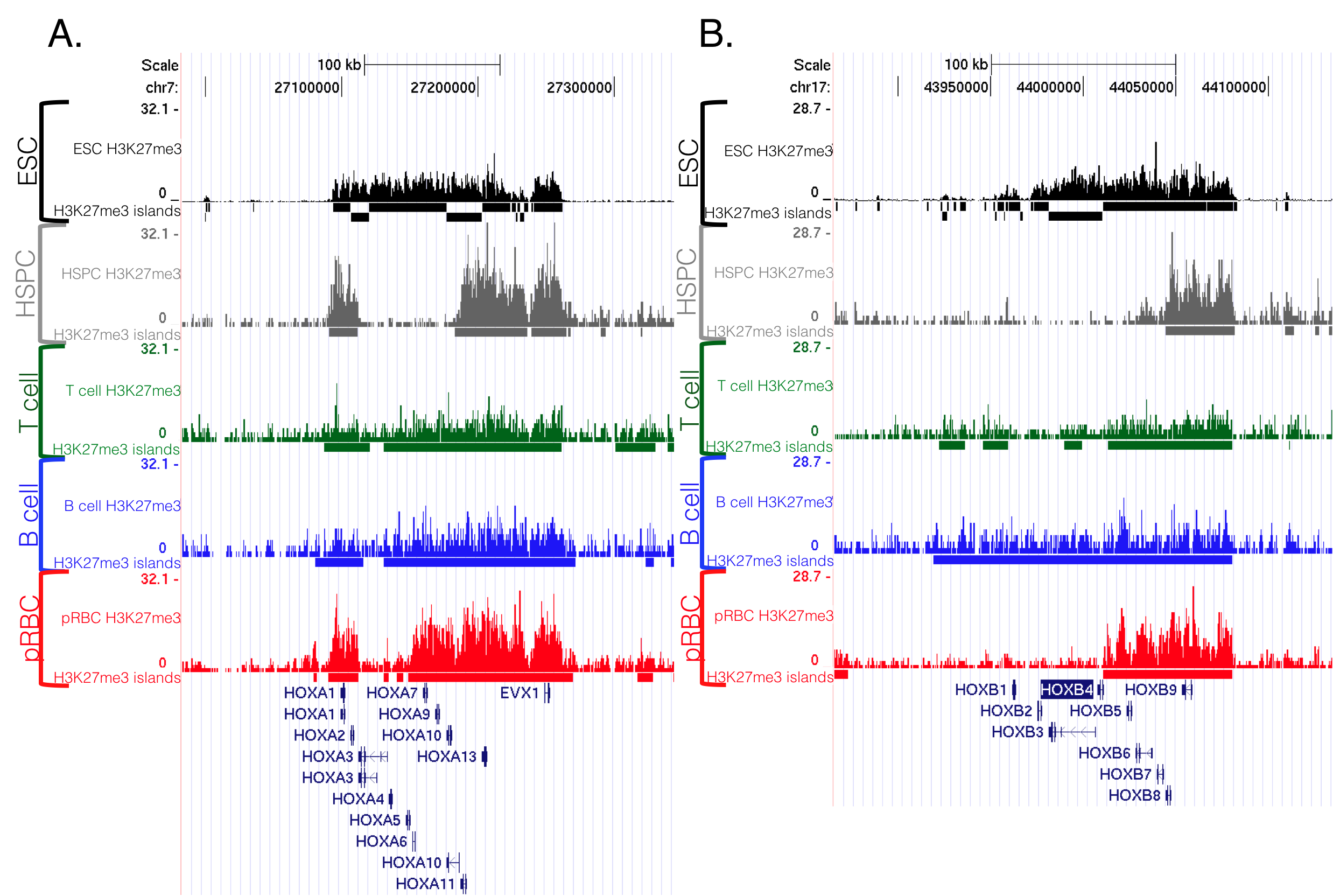
**

**Supplementary Figure S3A-S3B: Enrichment of H3K27me3 at *HOXA* and *HOXB* loci** – related to Figure 3.

1. H3K27me3 read density and statistically enriched SICER islands at the *HOXA* locus. The *HOXA* locus is silenced by H3K27me3 in ESC, partially loses H3K27me3 in HSPCs, and regains H3K27me3 enrichment in downstream cell types.
2. H3K27me3 is lost at the *HOXB* locus between ESC and HSPC. Specific genes regain H3K27me3 in downstream cell types.

**
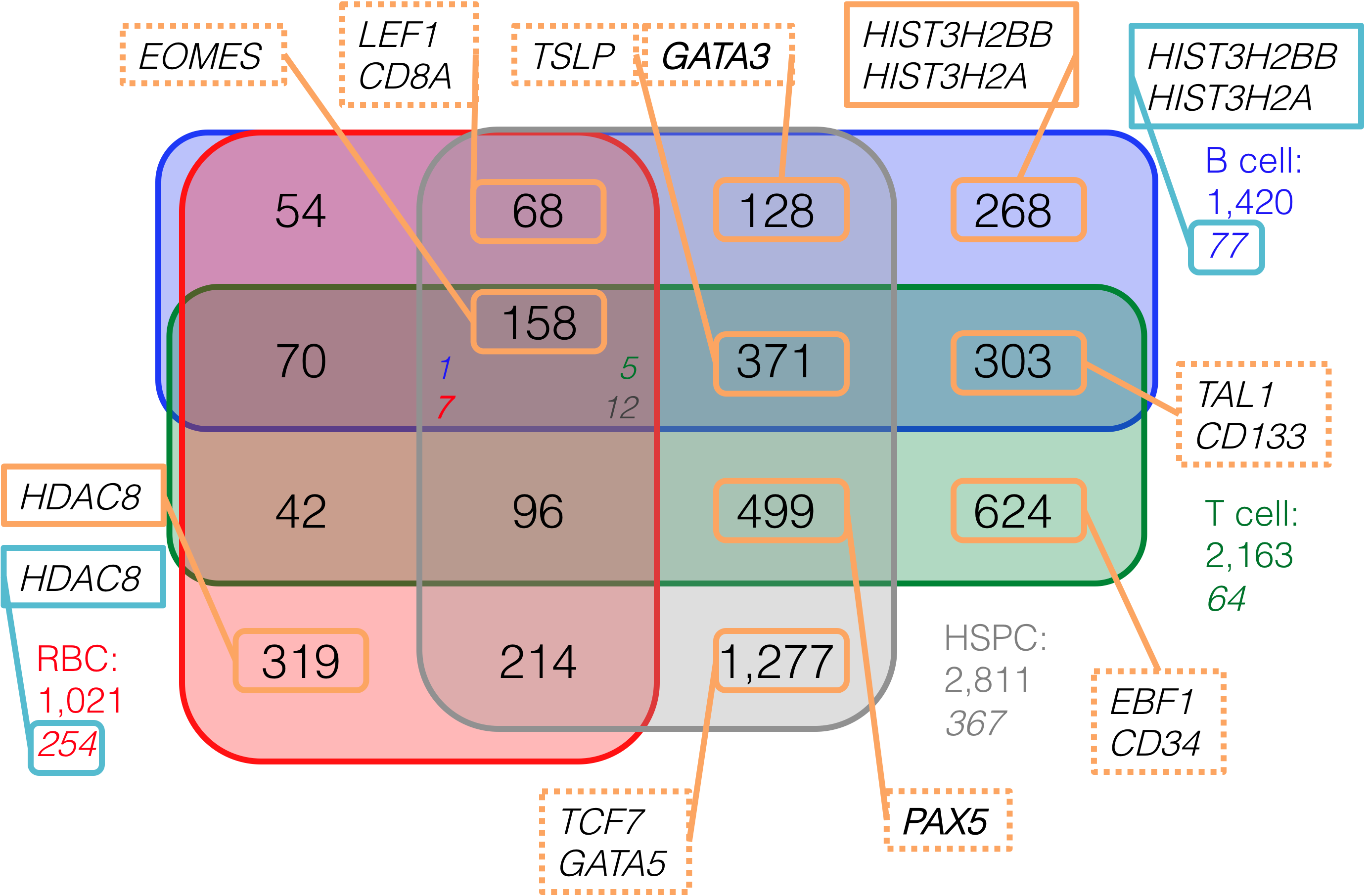
**

**Supplementary Figure 4: Bivalency of promoters across multiple cell types with examples** – related to Figure 3.

Total bivalent promoters in four cell types were counted, and those also bound by Pol II are noted in italics. Genes with bivalent promoters in combinations of these cell types were counted and entered in a Venn diagram. Several important genes are highlighted. Promoters also bivalent in ESCs are outlined in a dashed line. Few promoters bivalent in all four cell types were also bound by Pol II in the cell types, as noted in their color in the intersection.

**
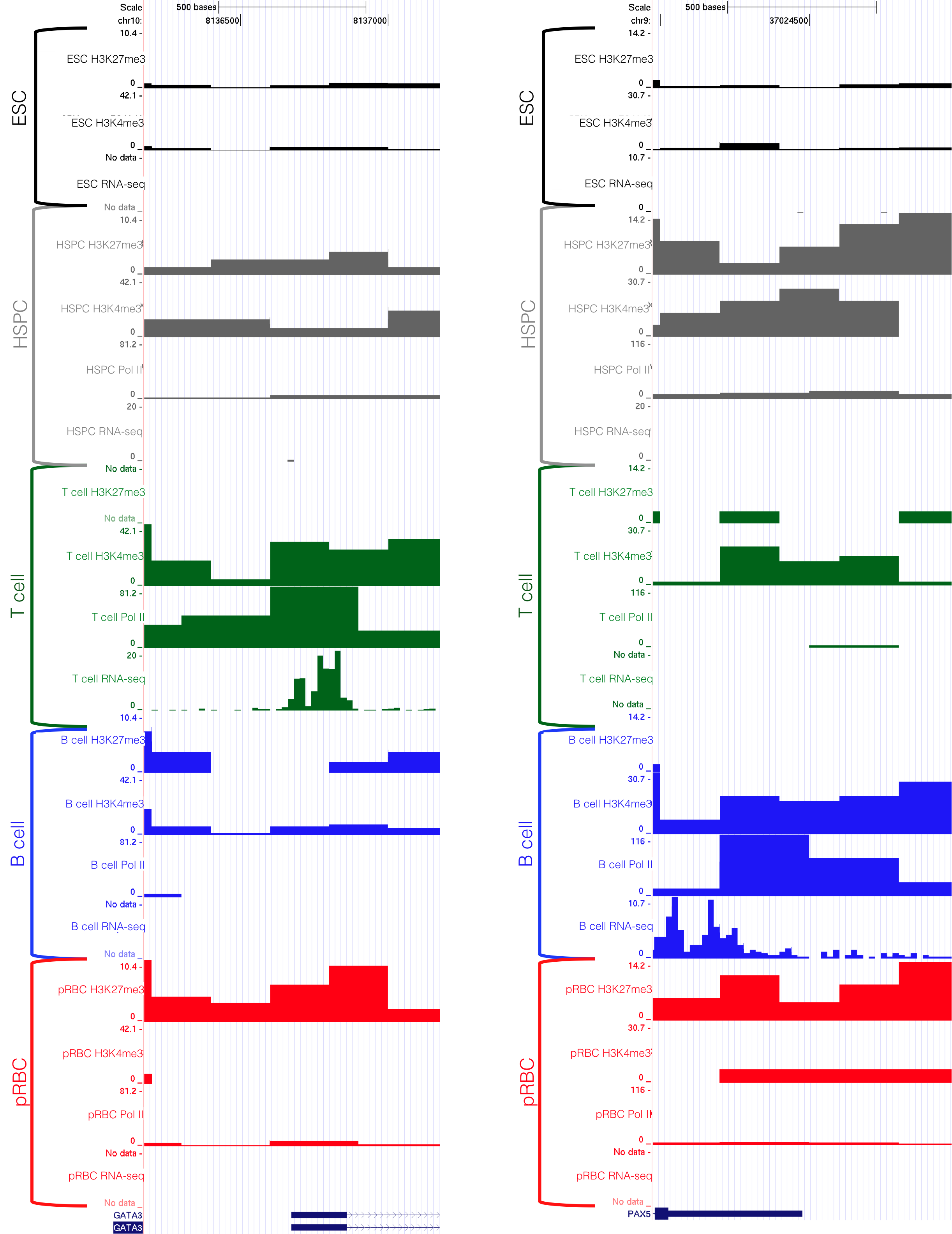
**

**Supplementary Figure S5:** Density of chromatin proteins at *Gata3* gene – related to Figure 4. Histone mark reads were shifted +/- 75bp based on strand and sorted into 200bp bins. Pol II reads were shifted +/- 100bp based on strand and sorted into 300bp bins. RNA-seq reads were not shifted and sorted into 20bp bins.

**
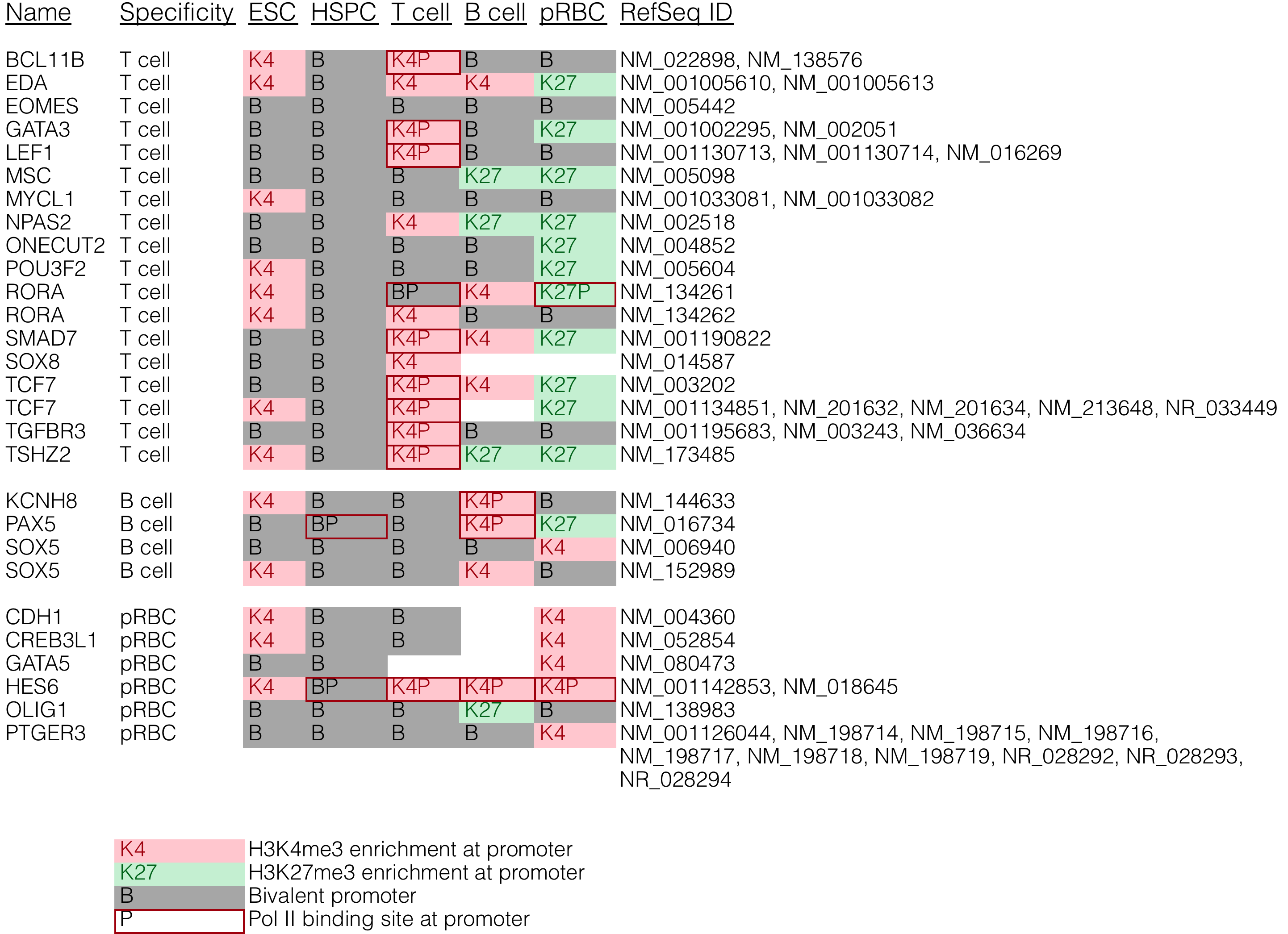
**

**Supplementary Figure S6: Resolution and preparation of transcription factor genes bivalent in HSPC** – related to Figure 3.

Promoters bivalent in HSPCs showing transcription factor activity by GO analysis, and type-specificity by their expression were interrogated in other cell types for their promoter enrichments in H3K27me3, H3K4me3, and/or Pol II. Few promoters were bound by Pol II in HSPC. Promoters tended to lose the H3K27me3 mark in their specific type and become bound by Pol II, but imperfectly. Many genes with multiple promoters resolved differently in the same cell type.

**Supplementary Table S1: Excel document of lists of type-specific genes**

**Supplementary Table S2: Known enhancers and their enrichments in H3K4me1 and/or H2A.Z in four cell types**

**
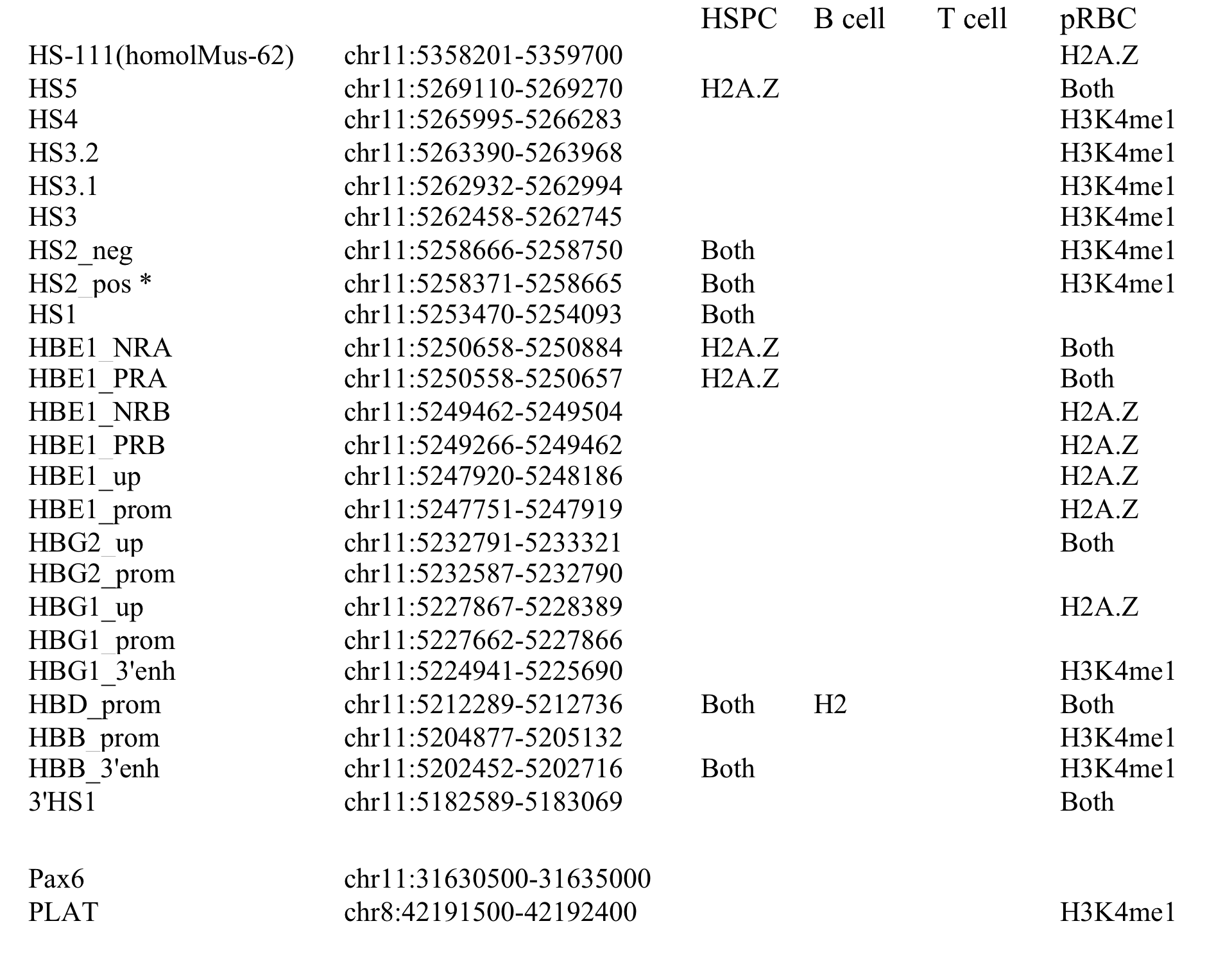
**

Hemoglobin enhancer loci defined in [^14^](#_ENREF_14)

RUNX1 intronic enhancer defined in [^15^](#_ENREF_15)

PAX5 enhancer defined in [^16^](#_ENREF_16)

PLAT enhancer defined in [^17^](#_ENREF_17)

**Supplementary Table S3: Files used in analysis and their sources**

**
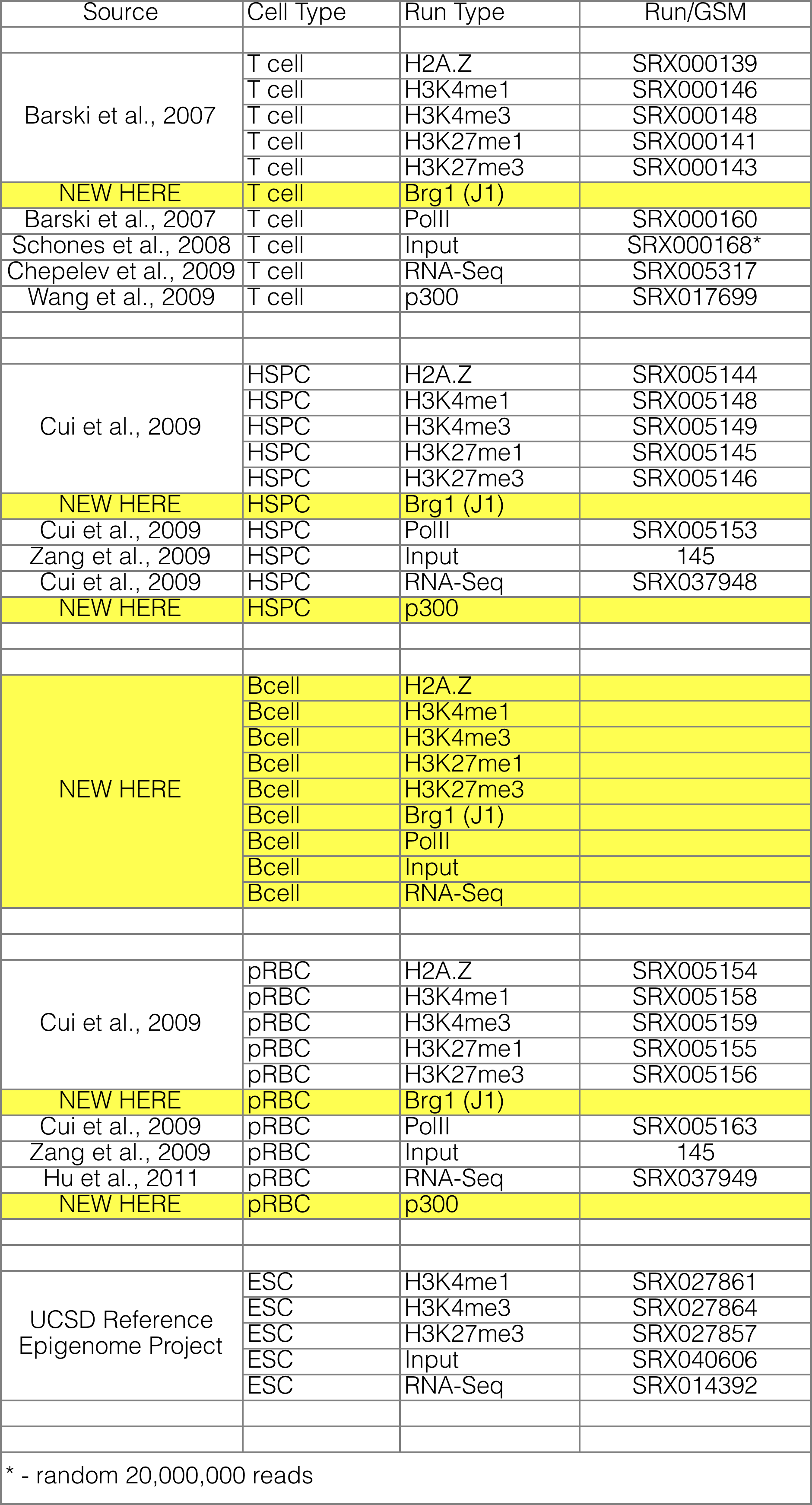
**

**Supplementary Table S4: Mapped sequencing reads, unique reads, and unique reads in enriched islands**. Unique reads allow one read per position.

**
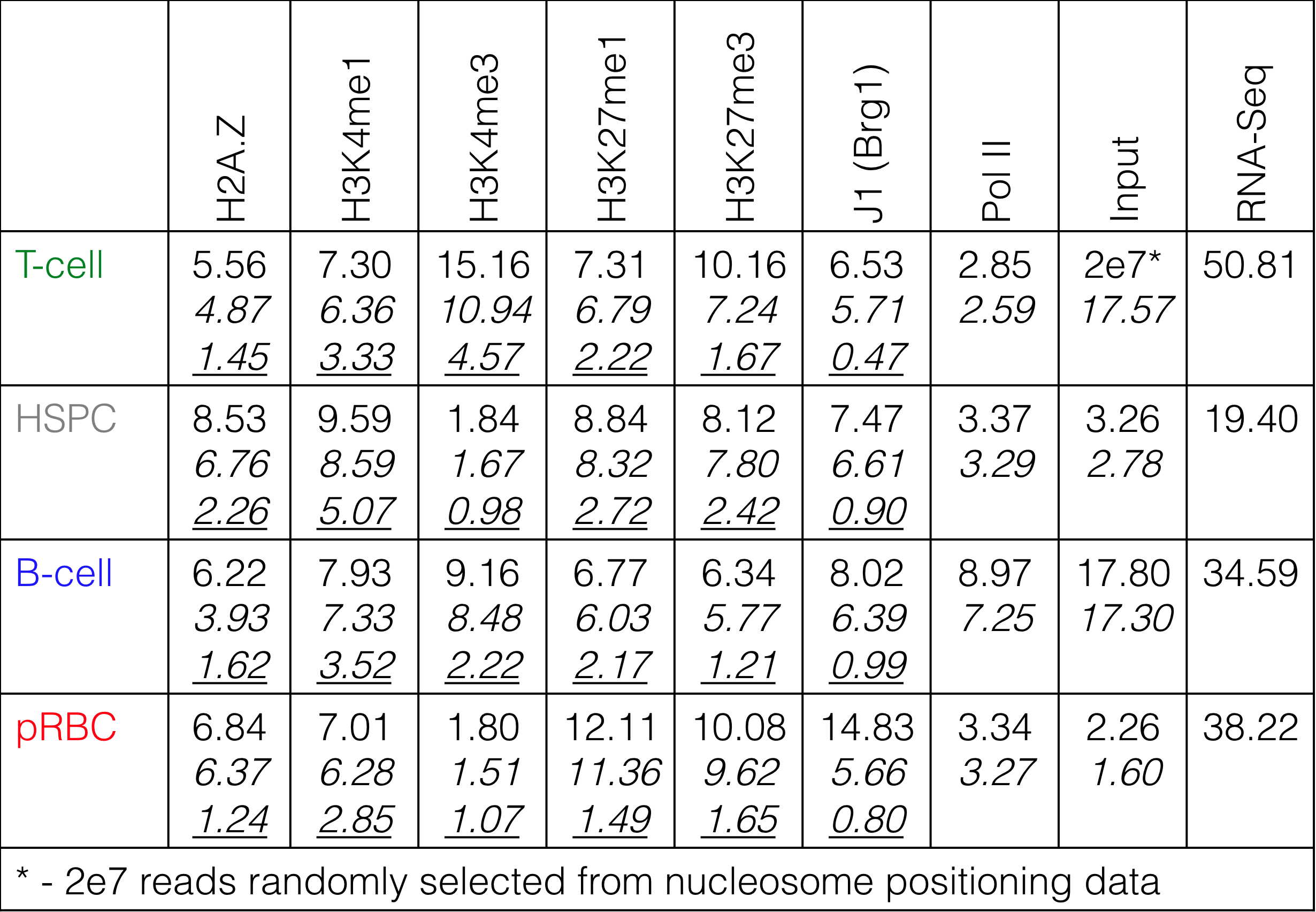
**

**4: Discussion of Figure 7B:**

Although enhancers have been predicted using a signature of H3K4me1 enrichment and depletion of H3K4me3 ([Heintzman et al. 2007](#_ENREF_28)), our data indicate that genes containing an H3K4me3-enriched CSPE showed higher expression than those merely containing a predicted regulatory element (p = 9.99x10^-9^), consistent with a previous finding that H3K4me3 is associated with active enhancers at a well-characterized genomic locus[^18^](#_ENREF_18). In contrast, genes associated with H3K27me3-enriched CSPEs showed lower overall expression (p < 2.2x10^-16^), as did genes associated with CSPEs that had an H3K4me3/H3K27me3 bivalent domain (p = 1.228x10^-3^), suggesting that these elements may serve as repressors of gene expression.

To find an explanation for the aforementioned enrichment of H3K27me1 at predicted enhancers, we compared the expression levels of genes associated with H3K27me1-enriched CSPEs with all genes associated with any CSPEs. Genes associated with H3K27me1-enriched CSPEs showed significantly lower expression than those with any CSPE (p = 1.776x10^-15^). However, they also tended to be significantly more highly expressed than the total gene list (p < 2.2x10^-16^). These results suggest that CSPEs, which are generally associated with higher expression, may not activate target genes when enriched in H3K27me1, suggesting that H3K27me1 could mark regulatory potential but not necessarily active transcriptional enhancement.

To test if combinations of H2A.Z and H3K4me1 more strongly predict functionality of the potential enhancer elements, we analyzed the distributions of genes associated with CSPEs defined by combinations of H2A.Z and/or H3K4me1 (**Figure 7B**). We noted that genes associated with CSPEs marked by either H2A.Z or H3K4me1 alone were expressed at lower levels than those associated with CSPEs defined by the union of the two modifications (CSPE vs. H3K4me1-only CSPE p<2.2x10^-16^; CSPE vs. H2A.Z-only CSPE p<2.2x10^-16^). The presence of both H3K4me1 and H2A.Z at the CSPEs was linked to higher expression of target genes than either H3K4me1 or H2A.Z alone (∩ CSPE vs. H2A.Z only p<2.2x10^-16^; ∩ CSPE vs. H3K4me1 only p=8.874x10^-13^). This indicates that the combination of H2A.Z and H3K4me1 is a better predictor of the functionality of the potential enhancers than either H2A.Z or H3K4me1 alone. We also tested if H3K4me3 at regulatory elements predicted by H3K4me1 enrichment alone has an impact on the expression of their nearby genes, and found that, in the absence of H3K4me3, CSPEs predicted by H3K4me1 alone were associated with significantly lower expression than all CSPEs predicted by H3K4me1 alone (H3K4me1 only vs. H3K4me1 & no me3 p=6.46x10^-4^), indicating that enhancers marked by H3K4me3 may be more active than those without H3K4me3.

1. Trapnell C, Pachter L, Salzberg SL. TopHat: discovering splice junctions with RNA-Seq. *Bioinformatics*. 2009;25(9):1105-1111.

2. Li H, Handsaker B, Wysoker A, et al. The Sequence Alignment/Map format and SAMtools. *Bioinformatics*. 2009;25(16):2078-2079.

3. Hu G, Schones DE, Cui K, et al. Regulation of nucleosome landscape and transcription factor targeting at tissue-specific enhancers by BRG1. *Genome Res*. 2011;21(10):1650-1658.

4. Robinson MD, McCarthy DJ, Smyth GK. edgeR: a Bioconductor package for differential expression analysis of digital gene expression data. *Bioinformatics*. 2010;26(1):139-140.

5. Kent WJ, Sugnet CW, Furey TS, et al. The human genome browser at UCSC. *Genome Res*. 2002;12(6):996-1006.

6. Williams T, Kelley C. Gnuplot 4.2: an interactive plotting program. 2011.

7. Saeed AI, Bhagabati NK, Braisted JC, et al. TM4 microarray software suite. *Methods Enzymol*. 2006;411:134-193.

8. Saeed AI, Sharov V, White J, et al. TM4: a free, open-source system for microarray data management and analysis. *Biotechniques*. 2003;34(2):374-378.

9. R Development Core Team. R: A language and environment for statistical computing. Vienna, Austria: R Foundation for Statistical Computing; 2010.

10. Koehler R, Issac H, Cloonan N, Grimmond SM. The uniqueome: a mappability resource for short-tag sequencing. *Bioinformatics*. 2010;27(2):272-274.

11. Zang C, Schones DE, Zeng C, Cui K, Zhao K, Peng W. A clustering approach for identification of enriched domains from histone modification ChIP-Seq data. *Bioinformatics*. 2009;25(15):1952-1958.

12. Shimazaki H, Shinomoto S. A method for selecting the bin size of a time histogram. *Neural Comput*. 2007;19(6):1503-1527.

13. Dennis G, Jr., Sherman BT, Hosack DA, et al. DAVID: Database for Annotation, Visualization, and Integrated Discovery. *Genome Biol*. 2003;4(5):P3.

14. King DC, Taylor J, Elnitski L, Chiaromonte F, Miller W, Hardison RC. Evaluation of regulatory potential and conservation scores for detecting cis-regulatory modules in aligned mammalian genome sequences. *Genome Res*. 2005;15(8):1051-1060.

15. Nottingham WT, Jarratt A, Burgess M, et al. Runx1-mediated hematopoietic stem-cell emergence is controlled by a Gata/Ets/SCL-regulated enhancer. *Blood*. 2007;110(13):4188-4197.

16. Decker T, Pasca di Magliano M, McManus S, et al. Stepwise activation of enhancer and promoter regions of the B cell commitment gene Pax5 in early lymphopoiesis. *Immunity*. 2009;30(4):508-520.

17. Heintzman ND, Hon GC, Hawkins RD, et al. Histone modifications at human enhancers reflect global cell-type-specific gene expression. *Nature*. 2009;459(7243):108-112.

18. Pekowska A, Benoukraf T, Zacarias-Cabeza J, et al. H3K4 tri-methylation provides an epigenetic signature of active enhancers. *EMBO J*. 2011;30(20):4198-4210.
